# Reporting Guidelines, Review of Methodological Standards, and Challenges Toward Harmonization in Bone Marrow Adiposity Research. Report of the Methodologies Working Group of the International Bone Marrow Adiposity Society

**DOI:** 10.3389/fendo.2020.00065

**Published:** 2020-02-28

**Authors:** Josefine Tratwal, Rossella Labella, Nathalie Bravenboer, Greet Kerckhofs, Eleni Douni, Erica L. Scheller, Sammy Badr, Dimitrios C. Karampinos, Sarah Beck-Cormier, Biagio Palmisano, Antonella Poloni, Maria J. Moreno-Aliaga, Jackie Fretz, Matthew S. Rodeheffer, Parastoo Boroumand, Clifford J. Rosen, Mark C. Horowitz, Bram C. J. van der Eerden, Annegreet G. Veldhuis-Vlug, Olaia Naveiras

**Affiliations:** ^1^Laboratory of Regenerative Hematopoiesis, Institute of Bioengineering and Swiss Institute for Experimental Cancer Research, Polytechnique Fédérale de Lausanne, Lausanne, Switzerland; ^2^Tissue and Tumour Microenvironments Lab, The Kennedy Institute of Rheumatology, University of Oxford, Oxford, United Kingdom; ^3^Department of Clinical Chemistry, Amsterdam University Medical Centers, Vrije Universiteit, Amsterdam Movement Sciences, Amsterdam, Netherlands; ^4^Section of Endocrinology, Department of Internal Medicine, Center for Bone Quality, Leiden University Medical Center, Leiden, Netherlands; ^5^Biomechanics Lab, Institute of Mechanics, Materials and Civil Engineering, UCLouvain, Louvain-la-Neuve, Belgium; ^6^Department Materials Engineering, KU Leuven, Leuven, Belgium; ^7^Laboratory of Genetics, Department of Biotechnology, Agricultural University of Athens, Athens, Greece; ^8^Institute for Bioinnovation, Biomedical Sciences Research Center Alexander Fleming, Athens, Greece; ^9^Division of Bone and Mineral Diseases, Department of Medicine, Washington University, St. Louis, MO, United States; ^10^Univ. Lille, EA 4490 - PMOI - Physiopathologie des Maladies Osseuses Inflammatoires, Lille, France; ^11^CHU Lille, Service de Radiologie et Imagerie Musculosquelettique, Lille, France; ^12^Department of Diagnostic and Interventional Radiology, Technical University of Munich, Munich, Germany; ^13^Inserm, UMR 1229, RMeS, Regenerative Medicine and Skeleton, Université de Nantes, ONIRIS, Nantes, France; ^14^Université de Nantes, UFR Odontologie, Nantes, France; ^15^Department of Genetics and Development, Columbia University Irving Medical Center, New York, NY, United States; ^16^Hematology, Department of Clinic and Molecular Science, Università Politecnica Marche-AOU Ospedali Riuniti, Ancona, Italy; ^17^Centre for Nutrition Research and Department of Nutrition, Food Science and Physiology, School of Pharmacy and Nutrition, University of Navarra, Pamplona, Spain; ^18^IdiSNA, Navarra's Health Research Institute, Pamplona, Spain; ^19^CIBERobn Physiopathology of Obesity and Nutrition, Centre of Biomedical Research Network, ISCIII, Madrid, Spain; ^20^Department of Orthopaedics and Rehabilitation, Cellular and Developmental Biology, Yale University School of Medicine, New Haven, CT, United States; ^21^Department of Comparative Medicine and Molecular, Cellular and Developmental Biology, Yale University School of Medicine, New Haven, CT, United States; ^22^Cell Biology Program, The Hospital for Sick Children, Toronto, ON, Canada; ^23^Maine Medical Center Research Institute, Center for Clinical and Translational Research, Scarborough, ME, United States; ^24^Department of Orthopaedics and Rehabilitation, Yale University School of Medicine, New Haven, CT, United States; ^25^Laboratory for Calcium and Bone Metabolism, Department of Internal Medicine, Erasmus University Medical Center, Rotterdam, Netherlands; ^26^Jan van Goyen Medical Center/OLVG Hospital, Department of Internal Medicine, Amsterdam, Netherlands; ^27^Hematology Service, Departments of Oncology and Laboratory Medicine, Centre Hospitalier Universitaire Vaudois, Lausanne, Switzerland

**Keywords:** bone marrow adiposity, bone marrow adipose tissue, bone marrow fat, marrow, adipocyte, standards, methods, Bone Marrow Adiposity Society

## Abstract

The interest in bone marrow adiposity (BMA) has increased over the last decade due to its association with, and potential role, in a range of diseases (osteoporosis, diabetes, anorexia, cancer) as well as treatments (corticosteroid, radiation, chemotherapy, thiazolidinediones). However, to advance the field of BMA research, standardization of methods is desirable to increase comparability of study outcomes and foster collaboration. Therefore, at the 2017 annual BMA meeting, the International Bone Marrow Adiposity Society (BMAS) founded a working group to evaluate methodologies in BMA research. All BMAS members could volunteer to participate. The working group members, who are all active preclinical or clinical BMA researchers, searched the literature for articles investigating BMA and discussed the results during personal and telephone conferences. According to the consensus opinion, both based on the review of the literature and on expert opinion, we describe existing methodologies and discuss the challenges and future directions for (1) histomorphometry of bone marrow adipocytes, (2) *ex vivo* BMA imaging, (3) *in vivo* BMA imaging, (4) cell isolation, culture, differentiation and *in vitro* modulation of primary bone marrow adipocytes and bone marrow stromal cell precursors, (5) lineage tracing and *in vivo* BMA modulation, and (6) BMA biobanking. We identify as accepted standards in BMA research: manual histomorphometry and osmium tetroxide 3D contrast-enhanced μCT for *ex vivo* quantification, specific MRI sequences (WFI and H-MRS) for *in vivo* studies, and RT-qPCR with a minimal four gene panel or lipid-based assays for *in vitro* quantification of bone marrow adipogenesis. Emerging techniques are described which may soon come to complement or substitute these gold standards. Known confounding factors and minimal reporting standards are presented, and their use is encouraged to facilitate comparison across studies. In conclusion, specific BMA methodologies have been developed. However, important challenges remain. In particular, we advocate for the harmonization of methodologies, the precise reporting of known confounding factors, and the identification of methods to modulate BMA independently from other tissues. Wider use of existing animal models with impaired BMA production (e.g., *Pfrt*^−/−^, Kit^W/W−v^) and development of specific BMA deletion models would be highly desirable for this purpose.

## Introduction

Bone marrow adipocytes (BMAds) reside in the bone marrow (BM) in close contact with bone, hematopoietic cells, marrow stromal cells, nerves, and blood vessels. Bone marrow adipose tissue (BMAT) thus refers to BM areas where BMAds are the predominant cell type, and BMA refers more broadly to BMAT across all skeletal locations and metabolic states. Over the last decades, interest in the functional role of BMAds has gradually increased and it is now evident that BMAds are actively involved in bone metabolism, hematopoiesis, and energy metabolism (1, 2). In addition, a possible role for BMAds in many diseases has emerged (3), and research groups all over the world are investigating the origin, function, and interaction of BMAds. However, different methods, models, and techniques are being used, which creates a challenge to compare or combine the results. Therefore, the International BMAS initiated a Methodologies Working Group to describe the existing methodologies, to identify associated challenges, and to establish standards in reporting as guidance for future studies in the field.

BMAT encompasses a heterogeneous population of mature adipocytes and preadipocytes, with distinct morphologies, lipid content, gene expression and function. Committed preadipocytes have a fibroblast-like morphology when observed *in vitro*, and are therefore morphologically indistinguishable from the progenitor populations encompassed within the term bone marrow stromal cells (BMSCs). However, preadipocytes are phenotypically very different from mature adipocytes. Preadipocytes are defined as cells committed through adipogenesis and characterized by the expression of early adipogenic genes (PPARy and CEBPa) (4, 5). Mature BMAds express late adipogenic genes (AdipoQ, Glut4, FABP4, LPL, PLIN1, ZFP423) and contain a single large lipid droplet, therefore resembling white adipocytes in appearance. In particular, adiponectin (AdipoQ) expression is already present in BM preadipocytes and stromal precursors, then increases with differentiation (6). Additionally, Krings et al. (7) have revealed that BMAT from whole tibiae in C57BL/6 mice possibly have a distinctive phenotype, expressing genes characteristic of both white and brown adipose tissue (WAT and BAT, respectively), congruent with the expression pattern of purified, primary human BMAds (7–9).

**Figure 1:**
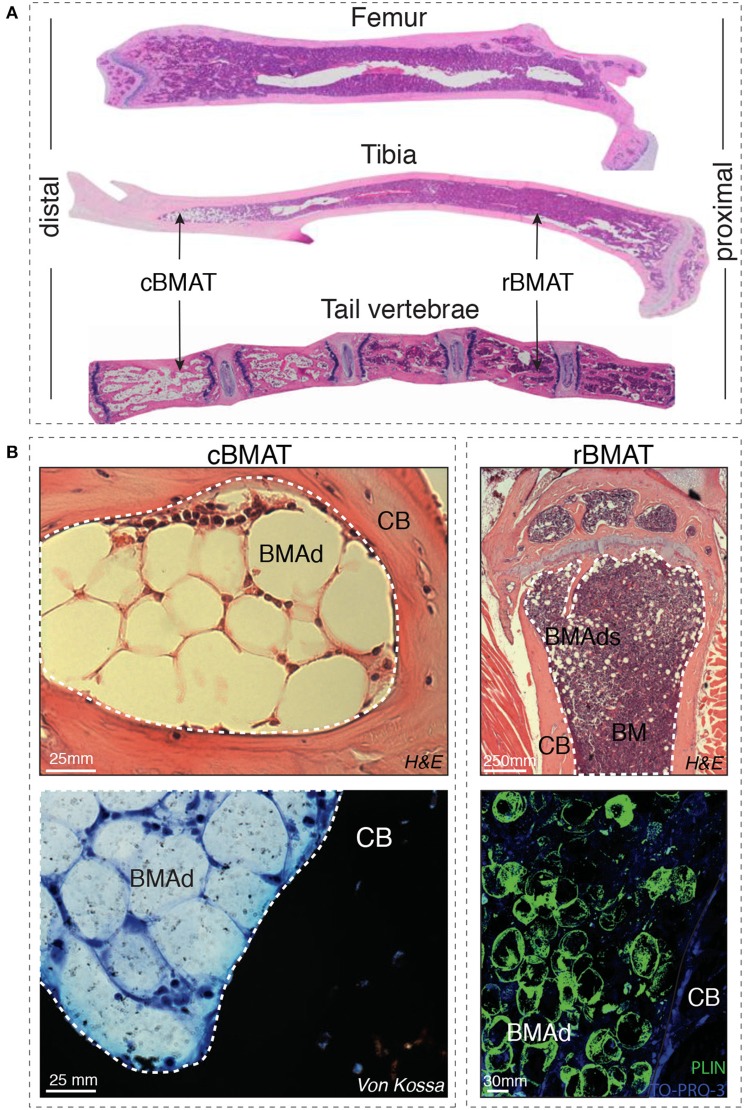
**(A)** Distal-proximal representative images in Hematoxylin & Eosin (H&E) stain of 4μm paraffin sections of femur, tibia and tail of 8-week-old mice. Note that the artefactually empty region in the center of tibia and femur corresponds to the expansion of the central vein lumen due to fixation-mediated retraction. **(B)** Left panels: Murine bone marrow adipocytes in the tail vertebrae of 24-week-old mice (cBMAT) of 3μm paraffin sections stained with H&E (top) and 6μm sections stained with von Kossa/Methylene Blue (bottom). Right panels: murine bone marrow adipocytes in the proximal tibia of 24 weeks-old mice (rBMAT) of 3μm paraffin sections stained with H&E (top) and 100μm sections stained with perilipin immunofluorescence (bottom, Perilipin in green and TO-PRO-3 nuclear counterstain) of 50-week-old mice. All images correspond to C57BL/6 female mice housed at room temperature fed *ad libitum* standard diet. BM, bone marrow; BMAd, bone marrow adipocyte; CB, cortical bone; cBMAT, constitutive bone marrow adipose tissue; H&E, hematoxylin and eosin; rBMAT, regulated bone marrow adipose tissue.

Indeed, Tavassoli et al. identified in 1976 two distinct populations of BMAds: after treatment with hemolytic anemia-inducing agent phenylhydrazine (10) one population remained stable while another population disappeared, and was described as labile BMAds. These two different “stable” and “labile” BMAd populations could be distinguished using performic acid Schiff (PFAS) staining (11). The presence of two different populations of BMAds localized to different regions of the skeleton was also more recently shown by Scheller et al. In mice, smaller BMAds (31–33 μm cell diameter) are interspersed between hematopoietic cells in the femur, the proximal portion of the tibia, and almost all skeletal segments that contain hematopoietic BM, while larger BMAds (38–39 um) are localized in the distal portion of the tibiae and phalanx [(12); Figure 1]. When challenged with cold exposure, BMAds interspersed in the red/hematopoietic marrow decreased in size and number, while the adipocytes localized in the yellow/adipocytic marrow did not change. The terms “regulated” and “constitutive” BMAT, respectively, have thus been proposed.

BMSCs and committed BM pre-adipocytes are more easily isolated and have seen a larger number of *in vitro* assays developed than mature BMAds, which are more difficult to handle in culture or to process in whole bone samples. *In vivo* lineage tracing models have started to pave the way, while specific markers for BMAd maturation remain to be identified. If successful, identification of specific biomarkers at the different stages of BM adipogenesis in both mouse and human will provide tools to dissect the impact of BMA in physiology and disease. *In vivo* imaging technologies are being adapted from studies of different tissues (e.g., peripheral adipose tissue) and species (e.g., human to mouse), while novel *ex vivo* imaging techniques are being adapted and developed specifically for BMAds and BM stromal imaging. All such techniques and their limitations and challenges are reviewed in the six sections that constitute this review, and guidelines for reporting of BMA-related results to maximize comparability are proposed in the concluding remarks (Table 1). For clarity, an abbreviation table is provided the manuscript (Table 2).

**Table 1:** Challenges and goals ahead for the BMA field and the BMAS WG in methodologies.

	**Challenges**	**Goals**
Main challenge	Standardize	Increase comparability by: • Homogenous definitions (c.f. BMAS consensus in nomenclature) • Homogenous reporting (c.f. BMAS Reporting Guidelines below) Increase reproducibility by: • Establishing consensus on standardized bone sites for analysis (e.g., rBMAT/cBMAT transition zones: tibia, caudal vertebrae) • Establishing consensus on reference groups • Establishing recommended standardized protocols: ° For *in vivo* modulation ° For *in vivo* extraction of primary BMAd and BMSCs ° For method-specific thresholds for BMA detection • Minimize effects of known confounding factors, to increase inter-study and multi-site comparison • Increase availability and accessibility of imaging techniques to implement use in routine clinical practice
Technical aspects	Adhere to minimal reporting guidelines	Implement the following **“BMAS Reporting Guidelines”**: • Specify precise BMA skeletal location in all figure legends • Report known confounding factors for all experiments: ° Skeletal location, gender, age, strain ° Ambient temperature (e.g., average housing temperature) ° Nutritional status (e.g., average food intake, antibiotics) ° Metabolic state (e.g., fasting, time of collection) ° Exercise (e.g., type of enrichment material in cages) • Report isolation technique with sufficient precision to reproduce; consider depositing protocol (e.g., protocol sharing platforms as recommended in the BMAS working group site at www.bma-society.org) • Report BMAd purity (e.g., hematopoietic/CD45^+^ cells, endothelial contamination, and CFU-F/BMSC) and viability/intactness • Report detailed imaging parameters, post-processing tools and algorithms
	Innovate	Development of: • Models for specific BMAd deletion • Specific BMA biomarkers • Recommended reference gene-set for adipogenic differentiation • Move from descriptive to mechanistic studies
Clinical perspectives	Define standards	Define the normal physiological values and increase functional understanding: • In humans by age, gender, skeletal location and lipid composition • In animal models by establishing a consensus reference group to be included as comparison in all animal studies (i.e., C57BL/6J 8-week-old female mouse as homeostatic control group)
	Disseminate	Facilitate access and use of unbiased BMA methodologies to non-experts (e.g., automated imaging, reference gene sets, reference cell trajectory maps)

*BMA, bone marrow adiposity; BMAd, bone marrow adipocyte; BMAS, Bone Marrow Adiposity Society; BMSC, bone marrow stromal cell; cBMAT, constitutive bone marrow adipose tissue; CFU-F, Colony Forming Unit-Fibroblast; rBMAT, regulated bone marrow adipose tissue*.

**Table 2:** List of abbreviations.

**Abbreviation**	**Definition**
2D/3D	Two/Three Dimensional
β3-AR	Beta-3 Adrenergic Receptor
Σ	Summation
μCT	Microfocus Computed Tomography
ACTH	Adrenocorticotropic Hormone
Ad.Ar	Adipocyte Area
Ad.Dm	Adipocyte Diameter
Ad.Pm	Adipocyte Perimeter
AdipoQ	Adiponectin
BADGE	Bisphenol A Diglycidyl Ether
BAT	Brown Adipose Tissue
BM	Bone Marrow
BMA	Bone Marrow Adiposity
BMAS	Bone Marrow Adiposity Society
BMAd	Bone Marrow Adipocyte
BMAT	Bone Marrow Adipose Tissue
BMD	Bone Mineral Density
BMFF	Bone Marrow Fat Fraction
BMSC	Bone Marrow Stromal Cell
BODIPY	Boron-Dipyrromethene
BV	Bone Volume
cBMAds	Constitutive Bone Marrow Adipocytes
CD	Cluster of Differentiation
COX	Cyclooxygenase-2
CR	Caloric Restriction
cAMP	Cyclic Adenosine Monophosphate
CEBPα	CCAAT Enhancer Binding Protein Alpha
CE-CT	Contrast-Enhanced Computed Tomography
CESA	Contrast-Enhancing Staining Agents
CFU-F	Colony-Forming Unit-Fibroblast
DAPI	4′,6-Diamidino-2-Phenylindole
DECT	Dual-Energy Computed Tomography
DIO	Diet Induced Obesity
DMI	Cocktail for Dexamethasone, IBMX And Insulin
EDTA	Ethylenediaminetetraacetic Acid
EGFP	Enhanced Green Fluorescent Protein
EM	Electron Microscopy
EU	European Union
FABP4	Fatty Acid Binding Protein 4
FACS	Fluorescence-Activated Cell Sorting
FBS	Fetal Bovine Serum
FDA	Food and Drug Administration
FSH	Follicle Stimulating Hormone
GDPR	General Data Protection Regulation
GFP	Green Fluorescent Protein
GH	Growth Hormone
Glut4	Glucose Transporter Type 4
H&E	Hematoxylin and Eosin
^1^H-MRS	Proton Magnetic Resonance Spectroscopy
Hf-WD-POM	Hafnium Wells-Dawson Polyoxometalate
HFD	High Fat Diet
HIV	Human Immunodeficiency Virus
Hm.Ar	Hematopoietic Area
HSL	Hormone-Sensitive Lipase
IBMX	Isobutylmethylxanthin
IGF-1	Insulin Growth Factor 1
IHC	Immunohistochemistry
ISCT	International Society for Cellular Therapy
Lepr	Leptin Receptor
LPL	Lipoprotein Lipase
Ma.Ar	Marrow Area
Ma.V	Marrow Volume
MAGP1	Microfibril-Associated Glycoprotein-1
MMA	Methyl Methacrylate
MR	Methionine Restriction
MRI	Magnetic Resonance Imaging
N.Ad	Adipocyte Number
NCD	Normal Chow Diet
ORO	Oil Red O
OsO_4_	Osmium Tetroxide
OVX	Ovariectomized
PLIN1	Perilipin 1
PCR	Polymerase Chain Reaction
PDGFRα	Platelet Derived Growth Factor Alpha
PDFF	Proton-Density Fat Fraction
PFAS	Performic Acid Schiff
Ppm	Parts Per Million
PPARγ	Peroxisome Proliferator-Activated Receptor Gamma
PRESS	Point-Resolved Spectroscopy
P/S	Penicillin/Streptomycin
PTH	Parathyroid Hormone
PTRF	Polymerase I and Transcript-Release Factor
rAAV	Recombinant Adeno-Associated Virus
rBMAd	Regulated Bone Marrow Adipocyte
RBC	Red Blood Cell Lysis
RFP	Red Fluorescent Protein
RNA	Ribonucleic Acid
RT-qPCR	Real-Time Quantitative Polymerase Chain Reaction
SFF	Signal Fat Fraction
SSC	Skeletal Stem Cell
STEAM	Stimulated Echo Acquisition Mode
SVF	Stromal Vascular Fraction
T.Ar	Tissue Area
TRAP	Tartrate-Resistant Acid Phosphatase
TV	Tissue Volume
TZD	Thiazolidinediones
UCP1	Uncoupling Protein 1
v/v	Volume/Volume
VMH	Ventromedial Hypothalamus
WAT	White Adipose Tissue
WFI	Water–Fat MR Imaging
WT	Wild Type
ZFP423	Zinc Finger Protein 423

Of note, even with such significant technological advances over the last decade, histological analysis has been a historical contributor in the understanding of BM composition and architecture, and is rapidly evolving through automatization via Digital Pathology. Histomorphometry therefore remains an important aspect of standard methodological practices in basic or translational research as well as clinical laboratories.

In addition, although rats and non-rodent animal models are recommended by the food and drug administration (FDA) or European Union as a model for osteoporosis, the field of BMA extends beyond bone health itself to the study of energy metabolism, hematopoiesis and metastatic bone disease, amongst other subfields. Mice constitute very important models for these other aspects of BMA research, especially in their quality of premier animal for genetic studies, and are thus recognized as preclinical model in this context. Nonetheless, we would like to encourage the study of BMA in larger animals and other rodents, especially when bone health and biomechanical properties of bone are being assessed.

## Histomorphometry

The field of bone histomorphometry was accelerated in 1976 when Dr. Parfitt published “Terminology and symbols in bone morphometry” which lay the foundation for the first Guideline on Bone histomorphometry (13). We can now build on this important consensus to establish additional guidelines on histomorphometry of BMAT. In 1987 Parfitt et al. listed three different meanings for bone; mineralized bone matrix, bone matrix, and bone tissue (14). Bone tissue encompasses bone and a soft tissue within it, the BM. The BM includes hematopoietic cells and its precursors, physically and functionally supported by diverse BM stromal cell populations [reviewed in (15)]. The latter is a three-dimensional network of cells in contact with developing blood cells in the extravascular space. The known main cell types that constitute this network are: osteogenic cells near bone surfaces, perivascular cells associated to sinusoids, and adipocytes. As discussed in this first section, methods to quantify marrow components via histomorphometry are based on different sample preparation, embedding and staining techniques, most requiring an intermediate step of decalcification and some allowing for epitope conservation for immunostaining. Paraffin-embedded samples have the advantage of access to large retrospective collections and potential comparability across sites, especially for the clinical setting where paraffin-embedding is standard. Other conservation procedures allow for more precise histomorphometric quantification, and some do not require decalcification [methyl methacrylate (MMA), or resin embedding, including technovit 900].

### Sample Preparation

Histomorphometric analysis relies predominantly on the quality of the sample. Therefore, careful consideration of the sample preparation is important. To prepare a BM sample, either calcified or decalcified bone samples can be embedded in paraffin or plastic, depending on the desired staining procedure (16, 17). For both procedures, the BM sample is regularly fixed in 4% Paraformaldehyde. Afterwards, the sample can be sectioned in conventionally 4–5 μm sections.

#### Decalcification

Several options of decalcifying agents are available, though Ethylenediaminetetraacetic acid (EDTA) is advised as compared to acid-based decalcification to enable most enzymatic and immunohistochemical stainings (18). Different factors can control the rate of the decalcification process: concentration of decalcifying agent, temperature, density of the sample, agitation and thickness of the tissue. In general, a large volume (e.g., 20x that of the sample), a high concentration of decalcifying agent and a high temperature (e.g., 20–37°C) during decalcification can speed up the reaction process. In contrast, increase of the size, density, and thickness of the sample may require longer decalcification time (17, 18). For an optimal immunostaining, an uniform decalcification of the sample is important, and we recommend decalcification at room temperature or 4°C on constant shaking, a large volume of EDTA to sample (at least 10:1 v/v) and several refreshments of the solution (every 3–4 days) to prevent calcium saturation (19).

Decalcification can also be performed using acidic agents to dissolve the calcium salts from the bone. This group of agents includes strong and weak acids. However, strong acid agents (nitric and hydrochloric acid) should be avoided in order to preserve the integrity of the cells and the enzymatic activity if subsequent immunostainings are desired (20). Among the group of weak acids (picric, acetic, and formic acid), decalcification performed with Morse's solution (50% formic acid and 20% sodium citrate) can also preserve the integrity of the sample for immunohistochemistry while allowing for high quality architectural evaluation with Hematoxylin and Eosin (H&E) staining (21). Dehydration is required prior to embedding. Ethanol dehydration in graded increases of ethanol and xylene, can allow for long-term storage of bones in 70% ethanol prior to embedding (22).

#### Embedding

To embed the samples after dehydration, several options exist. Most common and available is paraffin embedding. Decalcification is, however, necessary. This protocol is very useful to perform immunostaining, but the integrity of BM content, due to the juxtaposition of hard (decalcified bone) vs. soft (marrow) tissue is not guaranteed. Alternatives to paraffin are MMA or technovit 900 embedding. These do not require decalcification and allow for better preservation of the adipocyte morphology. However MMA and technovit 900 are less available in most laboratories and immunohistochemical staining becomes a challenge due to the destruction of the antigen presentation with the conventional MMA embedding protocols (23). With all embedding methods the histological procedure dissolves all the lipids in the vacuole, therefore the adipocytes are referred to as “ghosts,” which makes it impossible to investigate lipid content and composition in combination with histology.

To resolve this issue, Erben et al. developed an alternative protocol for plastic embedding, that avoids the complete loss of enzymatic activity in the tissue by adding methylbenzoate during the infiltration process and polymerization of the plastic (24). Here, cold embedding seems to be crucial for antigen presentation in the immunohistochemical procedure. Enzymatic activity is also preserved by using another resin embedding system (e.g., Technovit 9100) that contains methyl methacrylate and catalysators that allow the polymerization at low temperature (4°C) (25).

#### Staining

Although the adipocyte lipid vacuole is empty due to the ethanol-based dehydration necessary for histological procedures, these mature adipocyte ghosts are easily identifiable with several standardized staining procedures. The most frequently used in paraffin embedded bone is the H&E stain. Standardized staining procedures for plastic embedded bone, such as Goldner's trichrome, toluidine blue and Von Kossa staining can also be used to identify mature adipocyte ghost cells (24).

The discrimination between BMAds and blood vessels in a cross section can be difficult since the BM microenvironment is densely populated by blood vessels of different types and diameter and the endothelial wall is not always identifiable (26). Immunohistochemistry for Perilipin (27), a marker of mature adipocytes, is therefore useful for identification of BMAds in both human and murine tissues (Figure 1). Alternatively, immunostaining for Endomucin and/or CD31, markers for endothelial cells can be used to discriminate between blood vessels and adipocytes (28).

### Quantification

Two types of dimensional quantification are possible: two dimensional in terms of perimeter, diameter and area, and three dimensional in terms of volume and surface (29). Moreover, as described in the consensus on bone histomorphometry (30) and extensively discussed in the accompanying BMAS nomenclature position paper, BMA parameters should be presented in relation to a reference region (31). By using a common referent, it is possible to assess changes in the number or percentage of adipocytes following an intervention or comparing physiological and pathological states. For histological measures of BMAT, two-dimensional measurements of BMAT are applicable and two reference areas should be used: Marrow area (Ma.Ar) and total tissue area (T.Ar). It is important to distinguish between these two areas since the interpretation is notably different. When bone mass is lost and replaced by other marrow tissue, the Ma.Ar is increased while T.Ar remains similar. Marrow adiposity increases only when the area of BMAds increases relative to the marrow space. For three-dimensional *ex vivo* or *in vivo* measurement, Marrow volume (Ma.V) and Total tissue Volume (TV) should be used. A priori, two-dimensional measures should be used in standard bone histomorphometry and three-dimensional measures should be reserved for techniques which rely on 3D measurements, as discussed in the *ex vivo* or *in vivo* sections. Three-dimensional measurements may be used in histomorphometry when analysis of serial sections is performed to approximate volumes.

Additionally, measurement of the size of individual adipocytes is important in the analysis of BMAT, since the changes in total adipose tissue can be due to either an increase in the number of adipocytes or an increase in the size of the adipocytes. This distinction is important since the mechanism behind these changes can reveal both differences in adipogenic differentiation (affecting the number) or in lipolysis (affecting the size). In consensus with the Nomenclature working group of the BMAS, we suggest to use the terms Perimeter (Ad.Pm), Diameter (Ad.Dm), and mean Adipocyte Area (Ad.Ar) to address adipocyte size. Adipocyte areas can be reported for individual BMAds, giving rise to the frequency distribution of BMAd areas and corresponding measurement of mean or median Ad.Ar. In addition, adipocyte area can be reported at the tissue level as % of total adipocyte area relative to hematopoietic area (ΣAd.Ar/Hm.Ar), tissue area (ΣAd.Ar/T.Ar) or, most commonly, to marrow area (ΣAd.Ar/Ma.Ar) also commonly reported as “marrow adipose area” or less precisely as “marrow adiposity.” Hematopoietic area is defined either by CD45 positivity in immunohistochemistry or, morphologically, by the areas defined by the high density of hematopoietic cell nuclei within the marrow space. Exhaustive recommendations on BMA nomenclature are available in the accompanying white paper authored by the BMAS Working Group in Nomenclature (31).

Another important histological measure for adipocytes is the density of adipocytes. This is also used to differentiate between adipogenesis or enlargement of the adipocyte due to lipid storage. Adipocyte density can be measured as number of adipocytes per marrow area (N.Ad/Ma.Ar), number of adipocytes per hematopoietic area (N.Ad/Hm.Ar) or as number of adipocytes per tissue area (N.Ad/T.Ar). Adipocyte density varies greatly in the endocortical vs. trabecular regions of the bone, and thus detailed annotation and standardization of the quantified region is paramount, as detailed in the BMAS reporting guidelines (Table 1).

### Software

To quantify these parameters, a selection of software packages are available. Some have been developed for extramedullary adipose tissue and require manual adipocyte measurements, while others are designed for assessment of BM sections and are semi-automated, with a few entirely automated (listed in Table 3). Automated or semi-automated detection programs use shape (roundness, circularity) and the absence of color within the lipid vacuole for detection of BMAds. While such software packages are not yet routine, most laboratories have developed them in-house in order to perform adipocyte histomorphometry. Some of these software packages are freely available online (peerJ, fathisto, and MarrowQuant, see Table 3).

**Table 3:** Description of the most used software for bone marrow histomorphometry.

**Software**	**Species, sample**	**Automatic or manual**	**Other methods**	**Reported parameters**	**Proposed parameters**	**Other measures**	**Stains (embedding)**	**References**
OsteoMeasure	Monkey, proximal femur	Manual	–	–	Ad.Ar/T.Ar	–	–	(32)
OsteoMeasure	Human, biopsy	Manual	Blinded count	N.Ad, % Ad.V /TV (AV/TV); total Ad.Pm	ΣAd.Ar/T.Ar; N.Ad/T.Ar; total Ad.Pm	–	Goldner, 20x	(33)
OsteoMeasure	Mouse, femur	Manual	–	AV/TV; Ad.Pm; N.Ad/T.Ar.	ΣAd.Ar/T.Ar; Ad.Pm; N.Ad/T.Ar	Bone standard histomorphometry	Goldner's Trichrome/Von Kossa	(34)
OsteoMeasure	Mouse, distal femur	Manual	–	N.Ad/T.Ar	N.Ad/T.Ar	Bone standard histomorphometry	–	(35)
OsteoMeasure	Proximal tibia metaphysis	Manual	–	–	N.Ad/T.Ar	Bone standard histomorphometry	unstained (paraffin)	(36)
OsteoMeasure	Mouse, femur	Semi-automatic	–	Marrow fat content % (AV/TV)	ΣAd.Ar/T.Ar	Bone standard histomorphometry	H&E	(37)
OsteoMeasure	Mouse, tibia	manual	–	N.Ad/T.Ar; Ad.Dm	N.Ad/T.Ar; Ad.Dm	Bone standard histomorphometry	TRAP/toluidine blue	(38)
OsteoMeasure	Mouse, distal femur	Manual	–	Ad.Ar/T.Ar; N.Ad/T.Ar	Ad.Ar/T.Ar; N.Ad/T.Ar	Bone standard histomorphometry	TRAP/toluidine blue	(39)
OsteoMeasure	Mouse, distal femur metaphysis	Manual	Fat extraction and analysis	Ma. adiposity; Ad. Density	ΣAd.Ar/T.Ar; ΣAd.Ar/Ma.Ar; N.Ad/Ma.Ar	Bone standard histomorphometry	Methyl-methacrylate	(40)
OsteoMeasure	Human, bone biopsy	Manual	–	Ad.Ar/Ma.Ar	Ad.Ar/Ma.Ar	Bone standard histomorphometry	unstained, (plastic) 10x	(41)
OsteoMeasure	Mouse, tibia	Manual	–	N.Ad/T.Ar Ad.Ar/T.Ar	N.Ad/T.Ar; Ad.Ar/T.Ar	Bone standard histomorphometry	H&E	(42)
OsteoMeasure	Mouse, distal femur	Manual	–	Ad.V/TV	ΣAd.Ar/T.Ar	Bone standard histomorphometry	–	(43)
Image Pro	Mouse, distal femur	Manual	–	N.Ad; Ad.Ar	Ad.Ar/T.Ar; N.Ad/Ma.Ar	–	H&E	(44)
Image Pro Plus	Mouse, tibia	Manual	–	–	ΣAd.Ar/T.Ar; Ad.Ar/Ma.Ar; N.Ad/Ma.Ar	–	H&E	(45)
Image Pro Plus	Mouse, tibia	Manual	–	Ad.Ar	Ad.Ar/Ma.Ar	Bone standard histomorphometry	H&E	(46)
Image Pro Plus v6	Rat, femur	Manual	Manual count	N.Ad; Ad.Ar; Ad.Dm, Ad. density	ΣAd.Ar/T.Ar; ΣAd.Ar/Ma.Ar; N.Ad/Ma.Ar; Ad.Dm	Bone standard histomorphometry	H&E	(47)
Image Pro Plus v6	Rat, femur	Manual	–	Ad.Dm, N.Ad/Ma.Ar %Ad.Ar	Ad.Dm; ΣAd.Ar/T.Ar ΣAd.Ar/Ma.Ar; N.Ad/Ma.Ar	Bone standard histomorphometry	H&E	(48)
Image Pro Plus v6	Rabbit, distal femur	Manual	–	Ad.Dm; N.Ad/Ma.Ar; Ad.Ar/Ma.Ar	Ad.Dm; N.Ad/Ma.Ar; ΣAd.Ar/Ma.Ar	–	H&E	(49)
ImageJ	Rat, proximal tibia	Manual	–	Ad. content (Ad.Ar, T.Ar)	Ad.Ar; ΣAd.Ar/T.Ar	–	H&E	(50)
ImageJ	Mouse, femur or tibia	Manual	–	Ad.V/Ma.V	ΣAd.Ar/Ma.Ar	Bone standard histomorphometry	H&E (plastic or paraffin) 20x	(51)
ImageJ	Rat, proximal tibia	Manual	–	N.Ad/T.Ar; Ad.Ar/T.Ar	N.Ad/T.Ar; Ad.Ar/T.Ar	–	H&E	(52)
ImageJ	Mouse, distal femoral metaphysis	Automatic/ manual	OsteoMeasure	T.Ar adiposity, N.Ad/T.Ar, adiposity (%)	ΣAd.Ar/T.Ar; ΣAd.Ar/Ma.Ar; N.Ad/T.Ar; N.Ad/Ma.Ar	–	Von Kossa tetrachrome	(53)
OsteoidHisto	Human, biopsy	Semi-automatic	–	Ad.V/TV; Ad.V/Ma.V Ad.Dm N.Ad/Ma.Ar	ΣAd.Ar/T.Ar; ΣAd.Ar/Ma.Ar; Ad.Dm; N.Ad/Ma.Ar	Bone standard histomorphometry	Goldner's Trichrome	(54)
OsteoidHisto	Mouse, tibia	Semi-automatic	–	Ad.V/TV Ad.V/Ma.V Ad.Dm Ad.Dm	ΣAd.Ar/T.Ar; ΣAd.Ar/Ma.Ar; Ad.Dm; N.Ad/T.Ar	Bone standard histomorphometry	Calcein blue/TRAP	(55)
Bioquant Osteo	Human, biopsy	Semi-automatic	Blinded count	N.Ad, Ad. size,	N.Ad/Ma.V; ΣAd.Ar/Ma.Ar	Bone standard histomorphometry	–	(56)
Bioquant Osteo	Rat, tibia	Manual	–	N.Ad/TV	N.Ad/T.Ar	Bone standard histomorphometry	Goldner's trichrome	(57)
Bioquant Osteo II	Mouse, femur	No information	–	AV/TV	ΣAd.Ar/T.Ar	Bone standard histomorphometry	Goldner's Trichrome or TRAP	(58)
Leica Q-win Plus	Rabbit, vertebrae	No information	–	Ad.Dm; N.Ad/Ma.Ar	Ad.Dm; N.Ad/Ma.Ar	–	Oil-Red-O	(59)
MarrowQuant	Mouse, skeleton	Semi-automatic	Osmium tetroxide stain with μCT	T.Ar; Ma.Ar; N.Ad; Ad.Ar; ΣAd.Ar/Ma.Ar; Ad.V (μCT)	T.Ar; Ma.Ar; N.Ad; Ad.Ar; ΣAd.Ar/Ma.Ar; N.Ad/Ma.Ar	Bone Ar, hematopoietic Ar, vascular Ar	H&E (paraffin) 20x	(60, 61)
MarrowQuant	Mouse, tibia	Semi-automatic	–	T.Ar; Ma.Ar; Ad.Ar; ΣAd.Ar/Ma.Ar	T.Ar; Ma.Ar; N.Ad; Ad.Ar; ΣAd.Ar/Ma.Ar; N.Ad/Ma.Ar	hematopoietic Ar, vascular Ar	H&E (paraffin)	[Rojas-Sutterlin et al. as reviewed in (61, 62)]
Metamorph	Mouse, femur	Semi-automatic	–	Ad.Ar; N.Ad	ΣAd.Ar/T.Ar; N.Ad/Ma.Ar; Ad.Dm	–	H&E	(63)

*Ad., adipocyte; Ad.Ar, adipocyte area; Ad.Dm, adipocyte diameter; N.Ad, adipocyte number; Ar, Area; Ma., marrow; Ma.Ar, marrow area; AV, total adipocyte volume; H&E, hematoxylin and eosin; TRAP, tartrate-resistant acid phosphatase; TV, total tissue volume; Σ, summation; μCT, micro-computerized tomography*.

### Challenges and Limitations

Histomorphometric analysis is a very useful tool to determine the quality of bone and assess changes in the number and size of cells. One of the limitations is that histomorphometry is a time-consuming technique requiring microscopy, software and/or manual quantification. In addition, it is a technique that until now has relied on the interpretation of the single investigator, and therefore demands a solid quality control system. The detection of BMAds can be hampered by the close connection of adipocytes in yellow/adipocytic areas, making the separation and adequate counting of clustered adipocytes a big challenge, in particular if membranes are not intact. In mice, the number of adipocytes in long bones tends to be lower in sites of regulated BMAT, and thus separate adipocytes can be more easily discerned and counted in the red/hematopoietic marrow.

However, the distinction of adipocytes from small blood vessels can be a challenge, whether for manual quantification or automated algorithms, especially in the younger animals or in the context of marrow regeneration. Additional immunostains to discern microvasculature from adipocyte ghosts are thus highly recommended as a validation step. Moreover, one must keep in mind that histological sections are a cross-section of the bone/marrow organ and thus of the BMAd itself. In general, validation of automatic detection of adipocytes is not described, neither by presenting data on quality control measurements nor by comparison with a manual method. We therefore consider manual detection of adipocytes the gold standard for histomorphometry until automated software packages have been validated. Annotation and standardization of the quantified region, as well as reporting of the experimental parameters as detailed in the BMAS reporting guidelines (Table 1) cannot be emphasized enough. Additionally, correlation of histomorphometry findings with *ex vivo* or *in vivo* bone measurements of lipid content to calcified tissue constitutes a much-valued biological validation of findings. Finally, a recommendation on which bone may be considered as standard for reporting is premature, but we agree that choosing areas of transition between regulated and constitutive BMAT is most informative (e.g., tibia and/or caudal tail for mice).

## *Ex vivo* Whole-Bone Imaging

Histological slicing and histomorphometry remain the gold standard for the *ex vivo* evaluation and characterization of biological tissues in general, and BMAT in particular, by measuring adipocyte cell size and cell number. However, histological assessment (sectioning, staining, imaging, and analysis) remains a challenging, time-consuming, and often costly technique (29). Moreover, spatial patterns as well as the spatial inter-relationship between different tissues within one sample (for example BMAT in relation to bone and vasculature) can be inaccurate or impossible. To overcome some of the limitations of 2D analyses, several 3D imaging techniques have emerged to quantify the morphometry, spatial distribution of BMAT and its inter-relationship with other tissues in the marrow. In addition, mass spectrometry and high-profile liquid chromatography remain complementary standard methods to dissect lipid composition upon extraction (64, 65).

### Contrast-Enhanced Microfocus Computed Tomography

X-ray microfocus computed tomography (μCT) is a very powerful tool for 3D imaging of mineralized tissues (66). High-resolution μCT (<2 μm voxel size) and nanoCT [down to 150 nm (67)] scans are achievable and a high field of view to voxel size ratio can be obtained (68). While one of the biggest advantages of μCT is its non-destructive character, a considerable limitation of this technology is its lack of specificity for soft tissues. Phase contrast μCT is a possible solution, as it can be used to, for example, enhance edges, which allows a better visualization of soft tissues (69). Indeed, it provides information concerning changes in the phase of an X-ray beam that passes through an object. Moreover, it uses monochromatic X-rays, resulting in accurate measures of the attenuation coefficient, and thus enabling quantitative μCT imaging. This technique requires, however, highly dedicated hard- and software, and is not readily available. In addition, to the best of our knowledge, phase contrast imaging has so far not been used to visualize BMAT. Therefore, the focus of this review is rather on desktop single energy, polychromatic absorption contrast-enhanced μCT (CE-CT) imaging. Although having its limitations in the cone-beam shape of the X-ray bundle and the lower X-ray flux compared to synchrotron μCT, scanning times down to 15 min for high-resolution imaging can be achieved nowadays. For this kind of CE-CT, typically, there are two kinds of contrast agents used for the visualization of soft tissues: perfusion contrast agents, mostly used for *in vivo* or *ex vivo* indirect imaging of vasculature, and contrast agents that bind to the tissues for *ex vivo* imaging, further referred to as contrast-enhancing staining agents (CESAs). Here, we will focus on CESAs, which have proven to allow CE-CT imaging of BMAT.

The introduction of CESAs has enabled contrast-enhanced CE-CT to become a very important tool in biomedical imaging. CESAs bind to tissues of interest, increasing the X-ray attenuation coefficient (70). The very first reports on the use of CESAs for CE-CT imaging of soft tissues go back to only about a decade ago. Indeed, several groups (71–73) used osmium tetroxide (OsO_4_) on mouse embryos, pig lungs, and honeybees, respectively, to enable virtual 3D anatomical analyses using CE-CT. Although in these studies OsO_4_ was used for general tissue staining, it is well-known for its specific binding to unsaturated lipids (74, 75). Consequently, several years later, Scheller et al. reported the use of OsO_4_ for 3D CE-CT visualization of BMAT and quantification of its amount and distribution in long bones of mice using standard μCT (12, 76) and subsequently, ultrahigh-resolution μCT (77).

**Figure 2:**
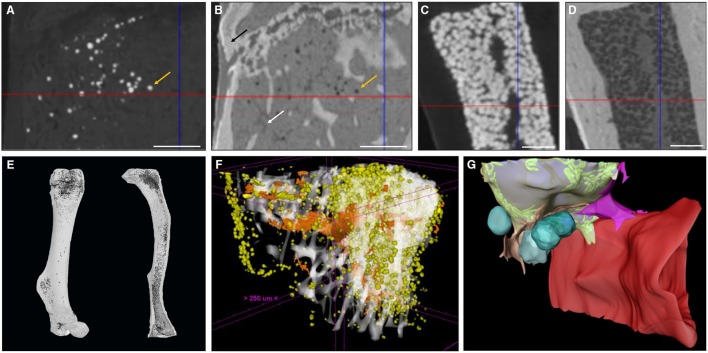
Zoom of a longitudinal CE-CT cross-section of the metaphysis of a murine tibia from a 16-week-old C57BL/6Rj male mouse fed *ad libitum* standard diet, using **(A)** osmium tetroxide and **(B)** Hf-WD POM staining, on the same sample. The orange arrows indicate the same adipocyte. The black arrow in **(B)** indicates the bone and the white arrow indicates a blood vessel. Zoom of a longitudinal CE-CT cross-section of the diaphysis of a murine long bone using **(C)** osmium tetroxide and **(D)** Hf-WD POM staining on the same sample. Scale bars = 250 μm. 3D rendering of **(E)** an osmium tetroxide stained murine femur (left) and tibia (right) from an 11-week-old C57BL/6J male fed *ad libitum* standard diet at room temperature, where adipocytes are presented in dark gray and bone in light gray. **(F)** Hf-WD POM stained murine tibia from a 30-week-old C57BL/6Rj male mouse fed high fat diet for 22 weeks [reprinted with permission from (78)], where white represents the bone, orange the blood vessels and yellow the marrow adipocytes. **(G)** 3D EM image of an adipocyte [reprinted with permission from (79)]. Lipid is shown in gray, mitochondria in green, cytoplasm in semi-transparent yellow, vascular sinusoid in red, perivascular cells in pink and orange, and blood cells in turquoise.

OsO_4_-based BMAT characterization requires a two-step scanning protocol: first, bones are detached and thoroughly cleaned from soft tissues, fixed, and scanned to enable characterization of 3D calcified bone parameters. The fixed bones are subsequently decalcified and then stained with OsO_4_ for 48 h (76) or longer if the mouse models develop severe BMAT accumulation. Subsequently, OsO_4_-stained bones are rescanned. These images provide 3D quantification of BMAT structural properties, such as adipocyte volume/total volume (Ad.V/TV), adipocyte volume/marrow volume (Ad.V/Ma.V), and adipocyte volume/bone volume (Ad.V/BV), which are quantified based on the amount of osmium-bound lipid. When combining OsO_4_ staining with high-resolution μCT imaging, individual adipocytes can be distinguished (Figures 2A,C,E) and a distribution of diameter can be calculated. When combined with image coordinate registration, this technique allows alignment of both the BMAT distribution and bone micro-architecture, as well as calculation of the distance of the BMAds from the bone surface (80). Some studies have also used this approach to measure BMAd density (cells/mm^2^ Ma.V) (81). The use of OsO_4_ for CE-CT-based BMAT visualization in mouse bones has quickly become widespread due to its compatibility with existing μCT infrastructure, ease of use, and reasonable cost (63, 82–84). As with most techniques, a high level of standardization is needed for each step in the procedure (fixation, decalcification, OsO_4_ staining, imaging, and analysis). For example, insufficient decalcification can lead to problematic osmium penetration and staining (85). Indeed, the limited tissue penetration capability makes staining of dense regions of adipocytes or larger bones problematic, restricting this technique primarily to whole bones in mice. Moreover, OsO_4_ staining is highly toxic, needing careful handling within a fume hood and appropriate disposal (86, 87).

To overcome these limitations, a recent study by Kerckhofs et al. reported the simultaneous visualization of mineralized and soft tissue structures within bones (Figure 2F) utilizing Hafnium Wells-Dawson polyoxometalate (Hf-WD-POM) as CESA (78). For this technique, murine long bones are incubated in POM powder dissolved in phosphate buffered saline while shaking gently for 48-h to 5-days. Samples are then scanned in the staining solution, or wrapped in parafilm and put in a sample holder for scanning. Thanks to the combination of the hydrophobic behavior of adipocytes and the binding of Hf-WD-POM to the BM tissue, visualization of the adipocytes is possible. When combining this CESA with high-resolution scanning (about 2 μm voxel size, maximum total image volume about 6 × 4.8 × 6 mm^3^), BMAds can be imaged at the single cell level (Figures 2B,D,F). This not only facilitates measurement of the volume fraction of BMAT within the bone (Ad.V), but also enables the quantification of the BMAd Number (N.Ad), density (N.Ad/TV), and Diameter (Ad.Dm). Additionally, with sufficient contrast, the vascular network can be discriminated from the other marrow tissues. This allows for full 3D blood vessel network assessment (i.e., branching and spatial distribution). Hence, Hf-WD POM-based CE-CT provides complementary data to standard histomorphometry, with enhanced 3D spatial information and inter-relation between different tissues in the BM compartment (Figure 2F). This was recently used to show that BMAT increased after menopause, and that increased BMAT was associated with osteoporosis and prevalent vertebral fractures (55). It should be highlighted that Hf-WD POM is non-invasive and non-toxic, and does not interfere with subsequent histological processing and immunostaining. A limitation of Hf-WD POM, however, is that it is not yet commercially available, although it can be requested in the frame of a collaboration.

When making a direct comparison between Hf-WD POM and OsO_4_ using high-resolution CE-CT, it was observed that both CESAs performed equally well for detecting BMAds (Figure 2). For locations with a low to medium BMAT amount, however, OsO_4_ staining was more sensitive in visualizing the sparsely distributed adipocytes (Figures 2A,B). For medium to high BMAT content, OsO_4_ tended to overestimate the adipocyte size due to high contrast difference between stained adipocytes and background, and thus contributed to the partial volume effect (Figures 2C,D). For this condition, Hf-WD POM allowed more accurate separation of individual adipocytes. Advantages and disadvantages of these *ex vivo* techniques are summarized in Table 4.

**Table 4:** Main quantitative parameters assessed when using *ex vivo* imaging techniques to explore bone marrow adipose tissue.

	**Parameters**	**Method**	**Advantages**	**Limitations**
2D techniques	**Histomorphometry**			
	• Adipocyte number (N.Ad) • Adipocyte size (Ad.V) • Adipocyte density (N.Ad/Ma.Ar) • Spatial localization (2D)	Resin, paraffin, or frozen sections (<5–10 μm)	• General availability • Can be used in all species • Pairs well with histological stains	• Slice/region bias • Limited field of view • Time consuming • High cost
3D techniques	**μCT—Osmium**			
	• BMAT volume (mm^3^) • BMAT density (%) • Adipocyte number^*^ (N.Ad) • Adipocyte size^*^ (Ad.V) • Spatial localization (3D)	• Whole bones or tissue samples, decalcified, and stained in osmium tetroxide solution. • Samples imaged and analyzed with μ- or high-resolution CT	• Adapts existing CT infrastructure and analysis techniques • Simple protocol • Low cost • Commercially available reagents • Highly sensitive for sparse BMAds	• Poor penetration in large, or high adiposity samples • Two-step scanning protocol for bone and BMAT analyses • Overestimates Ad.Dm • Highly toxic
	**μCT—POM**			
	• BMAT volume (mm^3^) • BMAT density (%) • Adipocyte number^*^ (N.Ad) • Adipocyte size^*^ (Ad.V) • Spatial localization (3D)	• Whole bones or tissue samples are immersed in POM solution. • Samples imaged and analyzed with μ- or nanoCT.	• Simultaneous visualization of bone, BMAT, and vessels in a single dataset^*^• Adapts existing CT infrastructure and analysis techniques • Simple staining protocol • Non-invasive • Accurate measure of Ad.Dm	• High spatial and contrast resolution needed to discriminate blood vessel network • Not yet commercially available • Not very sensitive for sparsely BMAds • Diffusion can take several days, depending on the sample size
	**FIB-SEM**			
	• Ultrastructure • Cell-cell interactions	3D electron microscopy	• Cellular and sub-cellular resolution of BMAd within niche	• Requires highly specialized equipment • Time-consuming • Limited field of view • High cost

*High-resolution μCT only (<2 μm resolution). Ad.Dm, Adipocyte diameter; N.Ad, Adipocyte number; Ad.V, Adipocyte volume; BMAd, bone marrow adipocyte; BMAT, bone marrow adipose tissue; FIB-SEM, Focused Ion Beam Scanning Electron Microscopy; POM, polyoxometalate; Ma.Ar, Marrow Area; μCT, micro-computed tomography.

### Future Challenges: 3D Microscopy

In recent years, standard microscopy techniques have also been optimized to gain 3D information about whole-bone cellular networks and nanoscale insight into the microenvironment of single cells. For example, tissue clearing strategies in skeletal tissues allow mapping of vascular networks and cell distributions in whole bones using light-sheet or two-photon microscopy (88, 89). Similarly, >50 μm-thick section immunohistochemistry can provide *ex vivo* insight into cell localization in 3D via conventional confocal microscopy (90). Though not yet published, we anticipate that clearing techniques described for marrow tissue will be used to provide novel information about BMAT localization and function. A key advantage relative to CT-based analyses is the ability to interrogate local cells and pathways that are defined based on expression of specific proteins and biomolecules using antibodies or genetically modified rodents.

At the nanoscale, focused ion beam scanning electron microscopy (EM), a form of serial EM that allows for 3D reconstructions at subcellular resolution, was recently applied to the BMAT adipocyte niche (79). This work builds upon previous 2D EM analyses of BMAT (11) and has helped to define interactions of BMAT with surrounding cells at the endothelial interface, within the hematopoietic milieu, and at the bone surface (Figure 2G). The major limitation of all of these techniques is the need for specialized imaging equipment. In many instances, data handling and analysis paradigms, which require very sophisticated statistical analysis to correct for the boundaries imposed by the confined bone architecture (91), are also just beginning to emerge. In any case, the development of regularly revised common standards and the commitment to BMAS reporting guidelines, as specified in Table 1, will increase comparability and pave the way for the comparative studies necessary to determine future gold standards in this rapidly evolving field.

## *In vivo* Imaging

**Figure 3:**
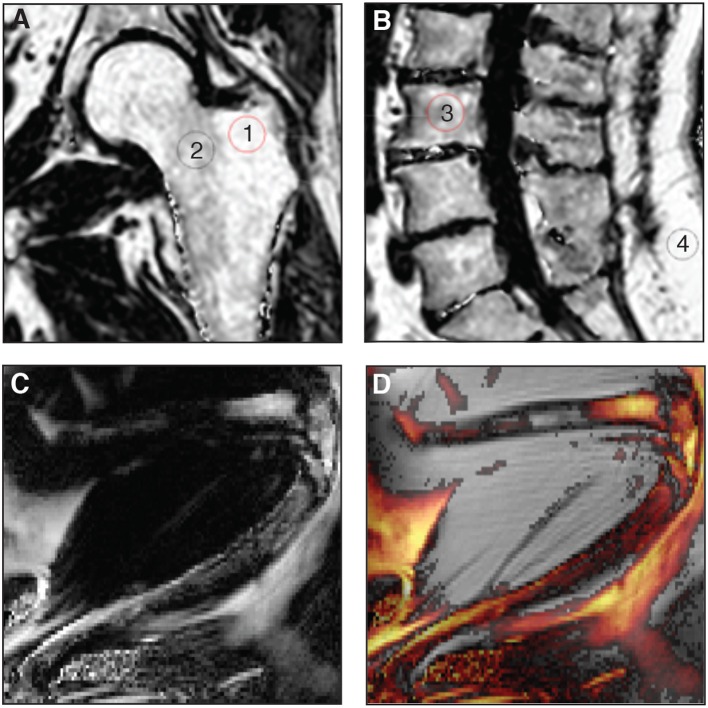
**(A,B)** Proton-density fat fraction (PDFF) maps generated using chemical shift encoding-based water-fat imaging from a commercially available sequence on a 3 Tesla magnetic resonance scanner. Coronal oblique acquisition of the left hip **(A)** and sagittal acquisition of the lumbar spine **(B)** of a 69-year-old woman with chronic lumbar and inguinal pain. Regions of interest can be drawn to assess bone marrow adiposity [(1) 94%, (2) 77%, (3) 71%, (4) 96%] at different anatomical sites through the PDFF parameter. **(C,D)** A three-point Dixon acquisition using a spin-echo based sequence and chemical shift encoding-based water-fat separation was conducted on a 9.4 Tesla horizontal magnet, to assess BMAT *in vivo* at the peak of aplasia after irradiation and bone marrow transplant in an 8-week-old C57BL6 female mice housed at room temperature fed *ad libitum* standard diet with antibiotics supplemented in the drinking water. The lower limb is shown as imaged in maximal flexion (61). Normalized fat content map **(C)** and fat content overlaid a magnitude image, red 10% - yellow 100% **(D)** show high BMAT content in the distal femur and also some BMAT in the proximal femur (horizontal, top of image) and throughout the tibia (diagonally across the image), in comparison to fat signal of surrounding extramedullary adipose tissue.

While *ex-vivo* techniques provide ample information of structures and allow for specific quantification of tissues, non-invasive imaging tools are essential when it comes to clinical studies so as to better understand the pathological processes that affect the BM *in situ*. To date, magnetic resonance imaging (MRI) is considered as the reference imaging modality to appraise *in vivo* BMA (92, 93). This powerful imaging tool has been used in animals, for example to follow the effects of zoledronic acid treatment on marrow adipogenesis in ovariectomized rats (47), to quantify the decrease in BMAT volume in obese exercising mice (63), and to follow the progression of BMA in murine hematopoietic recovery [(60, 61); Figures 3C,D]. Due to their small size, such measurements are not straightforward in rodents, as they require very strong magnetic fields for meaningful BMA signal detection, and dual-energy μCT is a valid alternative. MRI techniques are primarily applied *in vivo* in humans (Figures 3A,B). Indeed, the growing interest in BMA in relation with post-menopausal osteoporosis, fractures, metabolic perturbations, as well as over- or undernutrition states, opens up potential exciting perspectives for clinicians (94). However, the multiple interfaces between trabecular bone and bone marrow foster local magnetic inhomogeneities and challenge the accuracy and precision of BMAT quantification.

### What Can We Measure?

#### Main MR Imaging Biomarkers

The most relevant imaging biomarker used to quantitatively assess BMAT using MRI is the proton-density fat fraction (PDFF), which is the ratio of *unconfounded* fat signal to the sum of the *unconfounded* fat and water signals [(92, 92, 95, 96); Table 5]. As a result, the main challenge and limitation with quantitative BMAT assessment using MRI is to minimize the confounding factors to measure only signals coming from lipid protons. Interestingly, PDFF assessment of BMAT has benefited from technical developments in abdominal imaging. These technological improvements have been crucial for the emergence of reliable and non-invasive approaches to quantify adipose tissue in a standardized manner, especially through single-voxel proton spectroscopy (^1^H-MRS) and chemical shift encoding-based water-fat imaging (WFI) techniques (95).

**Table 5:** Main quantitative parameters assessed when using *in vivo* imaging techniques to explore bone marrow adipose tissue.

	**Parameter**	**Definition**	**Properties**	**Main imaging techniques**	**Outcome**
MRI- or CT-based techniques	Bone marrow fat fraction (BMFF)	Estimate of relative bone marrow fat content	• Generic term • Sensitive to experimental parameters when measured with MRI	• Single-voxel proton spectroscopy • Water-fat imaging • Dual-energy CT	Marrow fat content
MRI-based techniques	Signal fat fraction (SFF)	Ratio of fat signal to the sum of the fat and water signals	• Generic term • Specific to MRI techniques • Can be sensitive to MRI parameters	• Single-voxel proton spectroscopy • Water-fat imaging	
	Proton-density fat fraction (PDFF)	Ratio of *unconfounded* fat signal to the sum of the *unconfounded* fat and water signals	• Unconfounded imaging biomarker • Insensitive to MRI parameters	• Single-voxel proton spectroscopy • Water-fat imaging	
	Degree of lipid unsaturation	Ratio of signal coming from unsaturated lipids to the sum of all lipid signals	• Olefinic protons (5.31 ppm) are often used as an estimate of unsaturated lipids	• Single-voxel proton spectroscopy	Marrow fatty acid composition

*BMFF, bone marrow fat fraction; CT, computed tomography; MRI, magnetic resonance imaging; PDFF, proton density fat fraction; SFF, signal fat fraction*.

The second most common quantitative parameter reported in the literature reflects BMAT fatty acid composition. This specific evaluation is a topic of growing interest, as saturated fatty acids may have deleterious effects on the osteoblast lineage and may play a role in multiple inflammatory processes along with certain polyunsaturated fatty acids, affecting bone health (97). Fat composition assessment can be performed through an expression of its degree of unsaturation or polyunsaturation, calculated, respectively as the ratio of signal coming from the olefinic protons at 5.31 ppm or diallylic protons at 2.8 ppm on ^1^H-MRS acquisitions, to the sum of all lipid signals (Table 5), as discussed in detail in section Single-Voxel Proton Spectroscopy (93).

#### Robustness of ^1^H-MRS and WFI Methodologies

When the main biases are taken into account, WFI sequences appear to be robust in quantifying PDFF against changes in experimental parameters, in good agreement with ^1^H-MRS [*r* = 0.979 reported by (98), and *R*^2^ = 0.92 by (99)], using calibration constructs [BM phantoms: *R*^2^ = 0.97; (100)], and in agreement with histology using excised lumbar vertebrae [*r* = 0.72; (101)]. The intraclass correlation coefficients for repeatability and reproducibility of WFI were, respectively 0.997 and 0.984 (102), and the coefficient of variation in the quantification of PDFF varied from 0.69 to 1.70% (103). Moreover, a negative correlation (*r* = −0.77; 77) was demonstrated between ^1^H-MRS-based PDFF and *ex vivo* biomechanical vertebral properties (failure load), highlighting the relevancy of this parameter in bone strength (104). The reproducibility of ^1^H-MRS is known to be excellent, especially when assessing the lumbar spine *in vivo*, with an average coefficient of variation of vertebral bone marrow content of 1.7% (93, 105). Although ^1^H-MRS has long been considered the gold standard (105), WFI seems therefore to be a relevant and efficient alternative due to its ability to derive spatially resolved PDFF maps, with an absolute precision error of 1.7% between C3 and L5 vertebrae (106), and no significant differences with spectroscopic assessment in children (99) or in adults (98).

With regard to BMAT composition, although similar values have been reported between measurements from high-resolution proton spectroscopy acquisitions on *ex vivo* specimen and *in vivo* imaging (*R* = 0.61; 71), the true BMAT unsaturation level is consistently underestimated in *in vivo* acquisitions because of the fewer visible peaks. As a result, Li et al. preferred the use of *pseudo*-unsaturation level to better discriminate the apparent BMAT composition assessment in *in vivo* studies from *ex vivo* measurements. This differentiation in terminology reflects well the need to bear in mind the technical limitations encountered when evaluating fat composition *in vivo*.

### Toward a Better Standardization of MRI Techniques

Because ^1^H-MRS and WFI can be performed in most clinical facilities, their main technical limitations must be taken into account when assessing *in vivo* BMAT. A better standardization of the methodologies used to quantitatively assess BMAT would increase the accuracy of the reported PDFF in the literature, as well as the relevancy of inter-study comparisons.

#### Single-Voxel Proton Spectroscopy

Based on the frequency shift which exists between molecular groups, signals from water and lipid protons can be discriminated in a defined voxel of interest through 1H-MRS. However, although the area under each peak of the acquired spectrum is related to the number of protons of a specific chemical moiety, the MRS acquisition and post-processing analysis to calculate PDFF needs to consider the following confounding effects.

First, the water and fat components of BMAT have different T_2_ relaxation times. Therefore, in the absence of any T_2_-correction, the calculated signal fat fraction from ^1^H-MRS acquisitions is T_2_-weighted, depends on sequence parameters and overestimates the true PDFF. An ^1^H-MRS acquisition at different echo times combined with a T_2_ correction can removed T_2_-weighting effects (107, 108).

Second, even though initial ^1^H-MRS studies mainly considered the methylene group peak at 1.3 ppm to calculate bone marrow fat fraction or lipid/water ratio, adipose tissue has a complex spectrum made of multiple peaks. An oversimplification of the model used may reduce the accuracy of the qualitative and quantitative fat assessment. However, the trabecular microarchitecture promotes broad spectral peaks which make peak fitting challenging (93). Nevertheless, constrained peak fitting methodologies have been depicted and performed successfully at the hip and lumbar spine (107, 108).

Third, the short T_1_ value of bone marrow fat compared to water induces a relative amplification of the measured signal. PDFF calculations might be subsequently biased if T_1_ effects are not minimized. This effect can be minimized by using long repetition times for ^1^H-MRS acquisitions (93, 95, 109, 110).

Finally, the choice of the sequence mode is also of importance and depends on the employed echo times. By lowering J-coupling effects and being able to acquire spectra using shorter echo times, stimulated echo acquisition mode (STEAM) might offer a more accurate precise BMAT quantification compared to point-resolved spectroscopy (PRESS) sequences, despite its relatively noisier sensitivity (93).

The consideration of the above confounding effects is critical for assuring the robustness of MRS-based PDFF measurements across imaging protocols and imaging platforms, and essential toward the standardization of MRS-based PDFF measurements.

#### Water-Fat Imaging

##### Dedicated WFI techniques for BMAT assessment

WFI techniques share comparable confounding factors with ^1^H-MRS: there is a need for ${\text{T}}_{2}^{*}$ decay correction and T_1_ bias minimization, as well as a consideration of the multi-peak spectral characteristics of fat.

Indeed, due to the complex bone microarchitecture, the multiple interfaces between trabeculae and bone marrow induce an important but differential ${\text{T}}_{2}^{*}$-shortening effect affecting both water and fat. ${\text{T}}_{2}^{*}$ decay effects have therefore to be considered in the estimation of PDFF based on WFI. Despite its theoretical justification, a dual-${\text{T}}_{2}^{*}$ decay correction adjusting both water and fat relaxation times provides accurate bone marrow fat fractions at a nominal fat fraction close to 50% but noisy PDFF maps were reported in the spine in regions with lower values (104). Therefore, a single T2^*^ decay model should be at least adopted in BMAT WFI (93).

Regarding the T_1_-effect, the relative signal amplification can be easily lessened by using low flip angles or predetermined calibration values on WFI acquisitions (93, 95, 109, 110).

Finally, concerning the multi-peak spectral characteristics of fat, one direct consequence in WFI that illustrates an oversimplification of the model used is the “grayish” appearance of adipose tissues on water-only images generated from WFI reconstructions considering a single fat peak. This residual fatty signal may come from an incomplete discrimination of fat and water signals, especially between olefinic fat protons and water (111). Although this simplification is acceptable for most clinical applications, a more advanced modeling of the fat spectrum is necessary for a quantitative purpose.

##### Commercially available WFI solutions

As mentioned above, the methodological improvements of fat quantification using MRI emerged mainly from abdominal imaging. Most MRI vendors have played an active role in the development of these sequences. Although these commercial quantitative WFI solutions aim to quantify liver PDFF, these techniques may be an easier and interesting alternative to ^1^H-MRS in quantifying BMAT. An approximation with the multi-peak liver fat spectrum can indeed be considered, as only a negligible difference between the total signal fat from the 3 main peaks was reported when comparing the proximal femoral bone marrow and the liver (87 vs. 90%, respectively) (93, 107, 112). In addition, these sequences implement de facto a ${\text{T}}_{2}^{*}$ decay correction, and because the T_1_-effect can be simply lowered through a low flip angle, these solutions might be performed for BMAT PDFF assessment.

#### Other Technical Considerations

Other confounding factors may also be taken into account, such as noise-related bias (especially when using complex-based methods), eddy currents effects, gradient timing mis-registrations, phase errors in WFI and correction of J-coupling effects and chemical shift displacement effects in ^1^H-MRS (99, 107, 110). Their description goes beyond the purpose of this review, but they are fundamental for the development of future techniques.

### Current Challenges When Imaging *In Vivo* BMAT

#### A More Accurate Description of BMAT Fatty Acid Composition

Reporting of BMAT fatty acid composition through an expression of its degree of unsaturation constitutes the second most common quantitative parameter provided in the literature after PDFF-based quantification of total BMAT. Currently, only ^1^H-MRS can reliably assess BMAT composition.

However, contrary to PDFF assessment, there is not sufficient literature on the methodological considerations that should be followed in imaging studies. The importance of STEAM acquisitions over PRESS when assessing BMAT composition has been nevertheless highlighted, as a low reproducibility of unsaturation level measurements has been reported using the latter, with a reported coefficient of variation of 10.7% (105).

Moreover, the proximity and partial overlap of the olefinic peak with the water peak (present at 4.7 ppm) reduce the robustness of the peak fitting process. As a result, in addition to the previously described technical considerations, post-processing the spectra for this specific purpose is challenging, especially in young adults. Areas with low fat content, more frequently encountered in red bone marrow, limits the accuracy and precision of the reported measurements (93). The extraction of BMAT unsaturation levels is subsequently less prone to variations in yellow bone marrow or red marrow with elevated fat content.

Consequently, there is an urge to standardize and improve the reliability of this potential biomarker, as the degree of unsaturation of BMAT might have clinical implications, such as its potential role in the occurrence of fragility fractures (113, 114).

#### A Better Depiction of Physiological Values

To date, studies performed in healthy subjects have allowed for the description of physiological variations, especially in the spine. In children, WFI showed a decrease in PDFF measurements from the lumbar to the cervical spine, with a natural logarithmic increase with age but without sex difference (99). However, in adults, sex-related variations in addition to age-dependence of PDFF have been reported in the spine (106, 108). Regarding BMAT composition, differences in the degree of saturation have also been observed between adult males and females, with unsaturated lipids being higher in women (115).

Nonetheless, even though these physiological variations are critical for a better understanding of BMAT physiology, data is still insufficient in the literature to determine the exact normal values by age and gender, primarily due to the lack of standardization in methods used to assess BMAT.

#### Alternatives to ^1^H-MRS and WFI

Diffusion weighted-imaging, relaxometry, texture analysis, direct signal intensity, and dynamic contrast-enhanced imaging are alternative tools that have been performed to assess bone marrow adiposity. Although they provide interesting information, such as functional parameters related to bone marrow vascularization (116), these MRI techniques have not yet reached a consensus due to the insufficient number of relevant publications.

On the contrary, dual-energy computed tomography (DECT) is an emergent technique which may become a powerful alternative to MRI techniques as it can provide quantitative parameters representing both mineral and organic bone components (117). Consequently, whereas conventional single-energy quantitative computed tomography methods underestimate volumetric BMD measurements, DECT can correct for BMAT, resulting in more accurate densitometric measurements (118–120). Furthermore, BMAT content can be explored reliably, as good correlations have been reported with WFI and histology on cadavers (121, 122), and 1H-MRS *in vivo* (123). Potential interesting applications exist in oncology, to follow marrow fat expansion and BMD involution in patients after chemotherapy or radiotherapy (124). The main limitations of this modality are the radiation exposure, the need for prior phantom calibration, and the lack of standardization and data regarding reproducibility between different scanners and manufacturers.

In summary, MRI constitutes the current gold standard for *in vivo* imaging of BMAT in a clinical research setting, with current acquisition methods allowing for inter-center comparability. The field would however benefit from increased standardization, both in terms of reporting of confounding factors of the measured subjects as recommended in Table 1 and in terms of definition of standard sites of measurements, in order to increase comparability and to establish physiological reference ranges in humans and possibly larger mammals. The use of MRI for mouse models is only starting, due to the need for very strong magnetic fields for meaningful BMA signal detection; dual-energy μCT is a valid alternative for murine *in vivo* imaging.

## From Cell Isolation to *in vitro* Modulation

BMAds exist in a complex microenvironment within the bone, embedded within the marrow tissue where access to live cells for functional analysis is not trivial. Complementary to the above discussed challenges associated to BMAd imaging within their native environment, *in vitro* systems and *ex vivo* assays are crucial for understanding of the BMAd and its subtypes at the cellular level. The difficulty in isolating and handling primary mature adipocytes from the BM has led to the use of *in vitro* adipogenic differentiation assays from BM stromal cells as a surrogate method to study BMAd, an approach widely used in the field of peripheral adipocyte biology. This approach relies on the isolation of a stromal vascular fraction (SVF) from adipose tissue which is then plated and expanded in tissue culture plastic. The resulting adherent monolayer of stromal cells isolated from the BM, the so-called BMSC fraction, acquires the phenotype of multilocular and sometimes fully mature unilocular adipocytes in the presence of specific differentiation cocktails in standard 2D cultures. Mature BMAds, which *in vivo* develop only after birth (125), most likely originate from a specific subset of progenitor cells present within the BMSC fraction. This biological sequence thus supports, in part, the use of differentiated BMSCs to model BM adipogenesis. *In vitro* differentiation assays, however, reveal a cellular response to chemical stimuli resulting in a sum of specific phenotypes which describe *in vitro* plasticity, but do not necessarily reflect their native *in vivo* differentiation potential. The *in vitro* plasticity of BMSCs is in fact often larger than the plasticity revealed by *in vivo* readouts in native or injury-repair conditions, highlighting the importance of complementary *in vitro* assays and *in vivo* readouts to establish cell fate mapping within the BM, as described below and extensively reviewed elsewhere (126, 127). The sections below summarize key challenges and practical considerations to minimize variability and increase comparability in future BMAT cell-based studies, whether based on the isolation of primary BMAds or *in vitro* adipogenesis from BMSCs.

### BMAT: Location and Isolation

The BM is a soft tissue within the medullary cavity of compact bone. A mixture of hematopoietic precursors and differentiated cells, adipocytic cells, stromal cells, blood vessels, and nerve fibers occupy the marrow space within a complex network of extracellular matrix. Several techniques to isolate BMAT have been developed, all of which require invasive procedures to extract different populations from the encompassing bone.

In juvenile (age 8 to 12-week-old) mice, yellow/adipocytic marrow is present essentially in the distal tibia (filling up about one third of the total shaft length), the tail vertebrae and phalanx. Of the mouse strains systematically compared, BMAT is maximal in these locations in C3H/HeJ mice, and minimal in C57BL6/J mice (12). Older mice show a progressive increase in BMAT from distal to proximal, gradually gaining mature adipocytes in most skeletal sites of red/hematopoietic marrow. BMAT development and progression varies with strain and gender (128). Sites of murine BMAT for isolation in steady-state are thus small, and obtaining sufficient number of cells for cell sorting or cell culture purposes requires in most cases pooling samples from several animals.

It is important to note, as discussed in the *in vivo* modulation and *in vivo* tracing sections, that there may be differences in developmental origin according to the site of BMAT isolation. Due to the high degree of yellow/adipocytic marrow in the distal tibia, which also contains less trabecular bone than the caudal vertebrae, isolation of intact BMAT is relatively straightforward from the tibia after section at the epiphyses followed by gentle flushing or centrifugation. Contrarily, enzymatic digestion or mechanical disruption provides a higher yield of primary adipocytes from the tail due to the high number of caudal vertebrae and the predictable yellow/adipocytic marrow transition in the murine tail from the non-weight bearing segments. It is particularly important to note, however, that the fibrous tissue surrounding tail vertebrae is very rich in subcutaneous and periosteal adipocytes which require extensive mechanical removal or enzymatic digestion prior to isolation of BMAds to avoid contamination from subcutaneous adipocytes. One should also be aware that crushing bones can result in high cell death of BMAds and BMSCs, and it is thus to be avoided. Extraction of the intact BM plug or gentle mechanical disruption of the bone by fragmentation with a scalpel or scissors is thus preferred. Alternatively, a disrupted marrow plug can be obtained from smaller bones by removing the epiphysis and placing the open shaft in a PCR tube with a pierced bottom inside an Eppendorf tube containing a small amount of media (e.g., 200 μl), then gently centrifuging (e.g., 1 s at 500 g). BMAd markers are present in the top buoyant layer, while hematopoietic markers are only present in the pellet fraction upon RNA transcription analysis.

The long bones of mice are of similar size than human iliac crest biopsies, also called trephine biopsies, which involve the spongy bone and are performed for diagnostic purposes in hematology (1–2 cm long and 0.2–0.4 cm in diameter). Isolation and mounting approaches are thus often appropriate for both murine and human samples. Bone marrow aspirates are in most instances performed in parallel to trephine biopsies. In pediatric practice, BM aspirates are often performed from the sternum. Either are excellent sources of BMAT for research purposes after appropriate ethical approval. Debris from hip- or knee-replacement surgeries, including limb amputations, as well as spine neurosurgery also provide material rich in BMAds, as these are skeletal sites of abundant yellow/adipocytic marrow in the human adult.

Due to physiological BMAT specific variations according to species, strain, age, gender and skeletal site, as well as variations imposed by the isolation technique (flushing, spin-down, direct collagenase digestion, other enzymatic digestion), it is extremely important that researchers detail these parameters and indicate yield of primary BMAds or BMSC populations to favor comparisons across groups. Future efforts of the field should include evidence-based recommendations on extraction protocols that best preserve the heterogeneity of BMAd and their precursors. Other factors that may influence BMAT quality, and therefore yield, are related to body weight, bone weight or length and presence of metabolic perturbations or disease. BMAT obtained from human samples may be normalized to weight (μg) of tissue. Weight-based normalization remains however challenging for the small murine samples, where normalization per bone or “per leg” (e.g., tibia and femur) is standard (129).

### Mature BMAds: Isolation and Culture

Isolation of primary mature BMAds has been done to high purity by multiple gentle centrifugation steps (9, 56, 130, 131) and may include enzymatic digestion to aid dissociation of BMAds from their surrounding connective tissue. BMAds, just like visceral adipocytes, are fragile cells that are very sensitive to the strains of handling and temperature gradients. Samples must be manipulated gently and typically at 17–37°C to avoid lipid droplets from bursting. Generally, BMAd numbers obtained from murine bones are low due to the small volumes and their affinity to plastic and proneness to floatation or bursting, which encumbers handling. Cell counting of mature adipocytes by hemocytometer or flow cytometry is not representative of the sample at hand, and quality controls for purity and viability need to be devised through other methods including, for example, immunofluorescence for adipocyte yield and quantification of hematopoietic cell contamination (e.g., DAPI, phalloidin, LipidTox-DR and anti-CD45) or nuclei counting coupled to ceiling culture for quantification of yield of viable mature adipocytes. For claims on purified BMAds, especially those that refer to population based transcriptional analysis or proteomics/lipidomics, it is paramount that researchers specify the degree of hematopoietic and undifferentiated BMA cell contamination in both mouse and human BMAT. Single cell RNA sequencing techniques will facilitate BMAT studies, albeit at a high cost.

The possibility of fluorescence activated cell sorting (FACS)-based purification of mature adipocytes for downstream studies has been recently described for extramedullary adipocytes, based on forward/side scatter light signal and viability ensured by manual adjustments to the sorting pressure (132). It is to be demonstrated whether this approach may be compatible with primary BMAd isolation.

**Figure 4:**
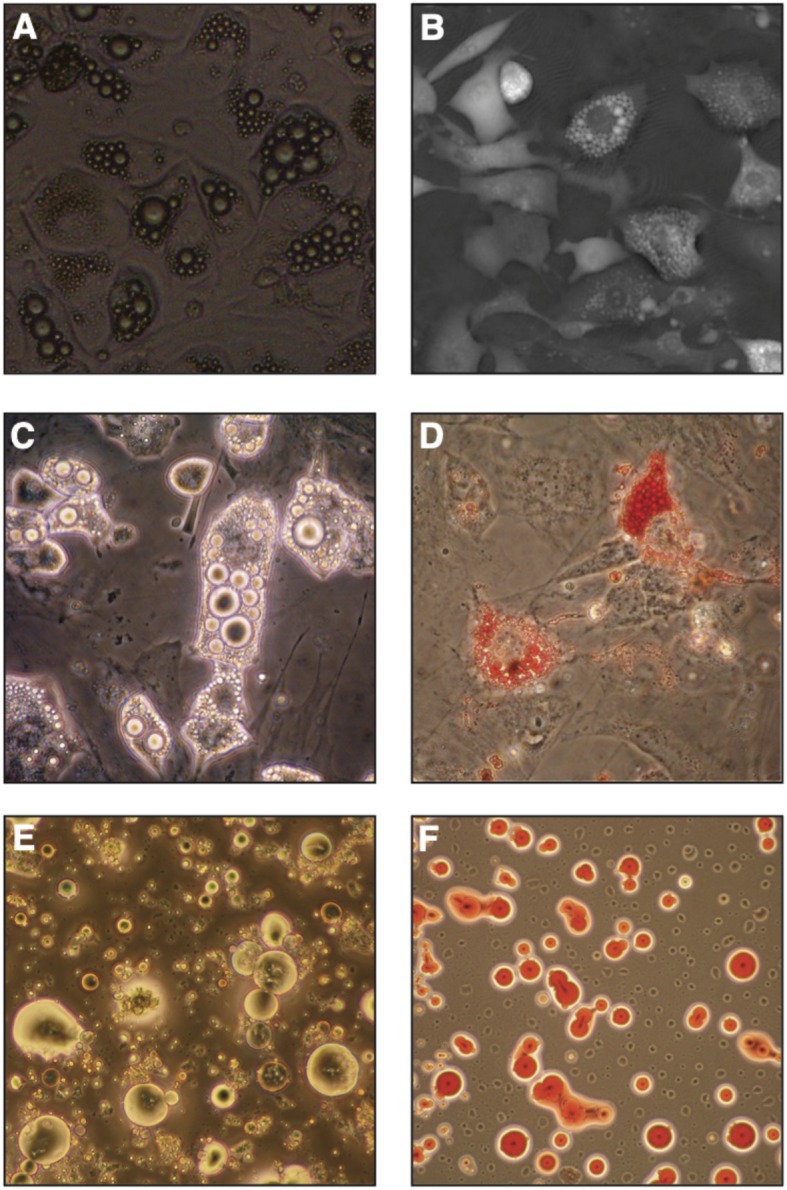
*In vitro* bone marrow adipocyte differentiation. **(A)** Bright field image (objective 10x) of OP9 cells differentiated in presence of serum, dexamethasone, insulin and IBMX (DMI cocktail) for 6 days, **(B)** Digital Holographic Microscopy (DHM) image of OP9 cells differentiated in DMI for 7 days. **(C,D)** Primary murine bone marrow stromal cells after *in vitro* differentiation in similar conditions imaged by light-transmission microscopy, where lipid droplets show a high refractive index **(C)** or stained with neutral lipid oil-soluble colorant Oil Red O **(D)**. **(E,F)** Primary murine BMAds from 2-month-old FVB female mice as seen by light-transmission microscopy **(E)** or stained with Oil Red O **(F)**.

Regardless of the isolation approach, a large number of primary BMAds must be initially isolated for most downstream assays (see section on BMAd assessment *in vitro*). On successful isolation of mature BMAds, their culture is delicate and short-lived. Ceiling culture in 2D allows for maintenance of BMAds for about 1 week (Figure 4E), after which de-lipidation is often observed. To avoid de-lipidation, irradiation of the cells has proven to be technically beneficial prior to culture (9). The recent description of protective 3D BMAd cultures in engineered devices or silk scaffolds holds great promise to recapitulate important clues for their behavior *in vivo* (133–135), but raises new challenges to develop efficient cell extraction protocols for endpoint analysis and 3D imaging techniques compatible with these set-ups.

### BMAd Progenitors: Isolation, Culture, and Modulation *in vitro*

Mature BMAds coexist hand-in-hand with their immature progenitors, which constitute a subset of the total BMSC fraction. The BMSC fraction has been defined by either (i) exclusion of endothelial and hematopoietic markers [typically CD31 to exclude endothelial components, CD45 to exclude hematopoietic components, and either murine Ter119 or human Glycophorine A to exclude nucleated erythroid lineage cells which lost CD45 expression, as discussed in Boulais et al. (136)], or (ii) by adherence and expansion in tissue culture plastic. Specific subpopulations with functionally validated *in vivo* stem cell or progenitor function, the so-called skeletal stem cells (SSCs) and their downstream committed or partially committed stromal, bone and cartilage progenitors have been recently described in mouse and human BM (137, 138). They present *in vitro* adipogenic potential and different degrees of *in vivo* adipogenesis, with human CD146 constituting the best functionally characterized marker for prospective isolation of SSCs (139, 140). Other skeletal multi-potent populations have been identified within the BM, including PαS (CD45^−^Ter119^−^PDGFRα^+^Sca1^+^) (141, 142), although care must be taken when defining clonal multi-potency (125, 140). Specific markers to prospectively isolate intermediate steps within the stromal to adipocyte commitment axis have also recently been identified in mice by Ambrosi et al. Namely, a tri-potent bone/cartilage/adipocytic perivascular CD45^−^CD31^−^Sca1^+^CD24^+^ stem-cell like population, a CD45^−^CD31^−^Sca1^+^CD24^−^ adipocytic progenitor population and a more mature CD45^−^CD31^−^Sca1^−^Zfp423^+^ BMAd precursor population were identified in the context of aging, high-fat diet (HFD) induced obesity and bone regeneration (5). No equivalent adipocytic differentiation hierarchy has yet been described in the human BM.

Due to the difficulty in isolating and expanding highly purified BMAd progenitors in mice, and to the lack of specific prospective BMAd progenitor markers in human BM, most studies to date have used unfractionated murine BMSCs, or *in vitro* expanded human BMSCs complying with International Society for Cellular Therapy (ISCT) standards (143) to produce *in vitro* differentiated BMAds for functional studies. ISCT standards provide a minimal set of surface markers and functional assays to validate human BMSC homogeneity. Standardized downstream functional assays have been proposed by the FDA (144–146). BMSC cultures rely on the rapid adherence of the cells to the culture dish, which allows exclusion of most hematopoietic cells from the culture. Nonetheless, passaging and sometimes sorting is necessary to eliminate macrophage contamination. As for primary isolated BMAds and to maximize comparability across studies, it is paramount to detail the source of BMSCs (gender, age, strain if applicable, metabolic diseases, skeletal location) and the method of isolation, such as specific enzymatic (e.g., collagenase-1,-2,-4, a combination thereof, trypsin) or mechanical dissociation as well as the specific expansion protocol, whose heterogeneity may explain some disparities in the field (summarized in Table 6, BMAS reporting guidelines as summarized in Table 1) (162). It is equally important to include quantification of contamination with hematopoietic or endothelial cells, and, specifically for BMSC populations, and to quantify the overall progenitor function of the primary isolate through fibroblastic colony forming unit assays (CFU-F) prior to adipocytic differentiation.

**Table 6:** Variability in murine bone marrow stromal cell isolation protocols.

**Samples**	**Isolation medium**	**RBC lysis**	**Enzymatic digestion**	**Depletion/enrichment**	**Application**	**References**
Flushed BM	DMEM/F12, 20% FBS, P/S, 2 mM L-glutamine, 0.1 mM NEAA, 3 mM sodium pyruvate	–	–	–	Cell culture	(147)
	PBS, 2%FBS, 2 mM EDTA	–	3 mg/ml col. I + 4 mg/ml Dispase (15 min 37°C)	–	Cell culture	(148)
	Leibovitz's L-15 medium, 1 mg/ml BSA, 10 mM HEPES, 1% P/S	0.8% NH_4_Cl	0.005% trypsin + 0.002% EDTA + 0.25 mg/ml col. IV (4 min 37°C)	Anti-CD45, Nestin-GFP+ FACS	Flow cytometry	(149)
	HBSS, 2%FBS	–	DNase I + col. IV *or +* liberase^DL^ (15 *or* 20 min 37°C)	–	Flow cytometry	(27, 150)
	DMEM, 15% FBS, 2 mM L-Glutamine, 1% P/S, 3.7 g/l NaHCO_3_	–	–	–	Cell culture	(151)
	α-MEM, 10% FBS, 1% P/S	–	–	–	Cell culture	(152)
	α-MEM, 15% FBS, 1% P/S, 2.2/l NaHCO_3_	–	–	–	Cell culture	(153)
	α-MEM	–	–	–	Cell culture	(154, 155)
	RPMI-1640, 10% FBS, 1% P/S	–	–	–	Cell culture	(156)
	-	-	Trypsin (2 min 37°C)	–	Cell culture	(84)
	Long term medium	–	–	–	Cell culture	(157)
	RPMI-1640, 20% FBS, 2 mM glutamine, 1% P/S	–	–	–	Cell culture	(158)
Centrifuged BM	N/A	RBC lysis buffer	–	–	BMAd isolation	(56)
	N/A	–	–	–	Cell culture	(135, 159)
Crushed long bones	PBS	–	col. (20 min 37°C)	–	Flow cytometry	(28)
Flushed and cut long bones	α-MEM, 10% FBS	–	1 mg/ml col. II (1–2 h 37°C)	–	Cell culture	(160)
Cut and washed long bones	DMEM	H_2_O 6 s	0.2% col. (1 h 37°C)	–	Flow cytometry	(141)
	N/A	–	5% col.	Anti-CD45, anti-Ter119, anti-CD31	Cell Culture	(161)
Cut long bones	PBS, 20% FBS	ACK	0.5% col. II (1 h 37°C)	–	Flow cytometry	(5)

*BSA, bovine serum albumin; Col., collagenase; DMEM, dulbeco's modified eagle medium; FBS, fetal bovine serum; RBC, red blood cell; RPMI, L-glutamine, phenol red, reduced serum; P/S, penicillin/streptomycin; NEAA, non-essential amino acids*.

Induction of adipogenesis from BMSCs *in vitro* has included a variety of inducers in standard 2D culture conditions, as summarized for primary murine samples in Table 7. Most differentiation techniques are based on methods developed for murine extramedullary pre/adipocytes (e.g., 3T3L1) or BMSCs primed for adipogenic differentiation (such as C3H10T1/2, 3T3-L1, or OP9). The common denominator includes a combination of the corticosteroid dexamethasone, which ultimately induces master transcriptional regulator of adipogenesis C/EBP-α, and phosphodiesterase inhibitor isobutylmethylxanthine (IBMX), which leads to cAMP accumulation, protein kinase A activation and thus PPAR-γ expression. The cocktail is classically accompanied by insulin exposure, whether from the serum or exogenously administered. Thus the acronym “DMI” cocktail for Dexamethasone, IBMX and insulin (163). In addition, adipogenesis can be further boosted through the use of cyclooxygenase-2 (COX) inhibitor indomethacin or PPAR-γ agonist rosiglitazone. Of note, the mechanical properties of the substrate are also determinant for BMSC differentiation, and even dominant to exogenous biochemical signaling (164), with softer matrixes favoring adipogenesis. The role of extracellular matrix components in this context, and its rate of degradation, has been however largely understudied in this context.

**Table 7:** Variability of *in vitro* murine bone marrow stromal cell adipogenic differentiation protocols.

**References**	**(147)**	**(84)**	**(156)**	**(160)**	**(****56****)** **(****135****)**			**(****5****)**	
**Maintenance**									
Medium	DMEM:F12	α-MEM	RPMI	α-MEM	α-MEM or DMEM			60% DMEM low Glc: 40%MCDB	
Serum	20% FBS	10% FBS	10% FBS	10% FBS	20% or 10% FBS			2% FBS	
Other	0.1 mM NEAA	200 μM NEAA						ITS, linoleic acid, dexa, AA, EGF, LIF, PDGFBB, bFGF	
**Adipogenic**									
Medium	DMEM:F12	α-MEM	DMEM	α-MEM	α-MEM or DMEM			60% DMEM low Glc: 40%MCDB	
Serum	20% FBS	10% FBS	9% horse serum	10% FBS	20% or 10% FBS			2% FBS	
Other		200 μM NEAA							
IBMX (μM)	500	500	450	0.5	500			0.5	
Dexa or Hydrocortisone (μM)	1	0.5	0.25	1	1			1	
Indomethacin (μM)	100	60						50	
Insulin (μg/ml)	5		5	0.01	10	10	10	5	5
Rosiglitazone (μM)			1		1	1			
T3 (nM)								1	1
**Differentiation time**	2 weeks	3 weeks	12 days	2 weeks	2–4 days	3–4 days	4 days	48 h	5 days

*NEAA, non-essential amino acids; dexa, dexamethasone; ITS, insulin-transferrin-selenium mix; AA, L-ascorbic acid 2-phosphate; EGF, epidermal growth factor; LIF, leukemia inhibitory factor; PDGFBB, platelet-derived growth factor BB; bFGF, basic fibroblast growth factor; FBS, fetal bovine serum*.

### BMAd Differentiation Assessment: *In Vitro* Assays and Applications

Multiple different cell types have the ability to accumulate lipid droplets, and thus we must evaluate the criteria with which we distinguish BMAds from other cells of the BM. In the context of extramedullary stromal differentiation, some groups have adopted the criteria of presence of at least four lipid droplets to define an adipocyte (165). This is especially useful as a threshold in imaging techniques where lipid droplets are visible (Figures 4A,C,E). As such, each investigator should critically evaluate what threshold is used as a definition.

Adipocytic differentiation is not completely efficient from primary BMSCs obtained on isolation, and the heterogeneity in cultures is well-known (166). This may be due to undetected heterogeneity of the initial BMSC population and adipocyte progenitors therein, to paracrine signaling cues in the culture, or, possibly, to presence of stromal cells that actively inhibit adipogenesis as recently described for CD142^+^ SVF cells in murine extramedullary adipogenesis (167). Moreover, as discussed above, *in vitro* differentiation potential may not faithfully reflect *in vivo* potential. Stringent *in vivo* assays in the form of heterotopic marrow formation by *in vivo* transplant in permissive conditions should thus be the norm to reveal the true lineage potential (140, 168, 169). Researchers must therefore rely on genetically modified mouse models with differential donor/recipient marker expression, or, in the case of human samples, in xenotransplants into immune-deficient mice with species-specific surface marker, Alu sequence or mitochondrial DNA detection to determine donor vs. host BMAds.

Upon isolation and culture or differentiation *in vitro*, assessment of BMAd maturation relies on the definition of the BMSC-to-BMAd axis and on established or forthcoming readouts. Classical biochemical techniques (including western blot, real-time qPCR, flow cytometry, RNA sequencing, lipidomics) require relatively large cell numbers, thereby limiting assay performance for BMAds. Importantly, the cells on each extreme of the maturation spectrum vary greatly, as simply illustrated by the morphological changes when comparing the spindle-shaped BMSCs with large lipid-filled BMAds (Figure 4). This must be accounted for in the selection of suitable references, such as reference genes for RT-qPCR that do not change upon adipocytic differentiation. Thus, cytoskeletal or metabolic genes must strictly be avoided as reference genes, while at least two early/mid- (PPARγ, CEBPα) and two late- (AdipoQ, Glut4, FABP4, LPL, PLIN1) stage markers should be quantified as genes of interest to cover the adipocytic maturation spectrum. The stability of reference genes needs to be demonstrated upon differentiation in every experimental setting, but others have identified good reference gene in the context of adipocytic differentiation from peripheral stromal cultures human and rodent studies (170, 171).

*In vitro* microscopy-based readouts classically detect lipid droplet formation with fluorescent dyes (e.g., Nile Red, ORO, BODIPY) (Figure 4F), or use of cells from fluorescently-tagged reporter mice (e.g., tdTomato, RFP, GFP as extensively reviewed in section *in vivo* Lineage Tracing). Whether for microscopy or flow cytometric applications, careful interpretation of results is required, as most mature BMAds will be lost on liquid handling, and care must be taken not to count lipid vacuoles from broken cells as BMAds. More recently, label-free techniques such as digital holographic microscopy (Figure 4B) or Raman-based microspectroscopy have been developed for *in vitro* BMAd cultures with high resolution and potentially improved performance over classical techniques (172, 173). By preventing staining and liquid-handling biases, these methods provide additional information on lipid content along with quantification of morphological parameters. Additionally, microspectroscopy holds the promise to reveal information on chemical composition at the single cell level, which may reveal physiologically relevant heterogeneity.

### Challenges in Cell-Based Assays

Isolation of primary BMAds remains challenging in both mouse and human. *In vitro* BMSC or BMAd precursor differentiation provides a valid alternative for studying the role of BMAds in cell-based assays, although potential differences with *in vivo* differentiated BMAds should always be acknowledged. This presents a challenge for normalization with age-matched control groups where the BMAds do not undergo similar changes. For appropriate normalization, it is thus important to account for both cell number and tissue weight, with pooling of control group mice to reach similar levels of BMAd isolation from the experimental and control groups for appropriate comparisons. For both primary BMAds and BMSCs, the cell mixtures obtained are highly dependent on the source and handling, and thus gender, age, skeletal location, metabolic perturbations, as well-extraction and culture methods should be thoroughly described as detailed in the recommended BMAS reporting guidelines (Table 1). With the application of *in vivo* BMA induction protocols (reviewed in Tables 8, 9), BMAds are modulated in cell size, number, and phenotypic/functional properties. Additionally, measures of BMAd purity and BMSC CFU-F progenitor function should be reported to increase comparability of results across different researchers. It is imperative that as the BMA field matures, so must the publication of consensus protocols as well as definitions for both BMAd and BMSC isolation and differentiation.

**Table 8:** *In vivo* modulation of bone marrow adipose tissue by dietary and environmental factors.

***In vivo* environmental intervention**	**Animal model**	**Outcomes (assay)**	**References**
High fat diet (45–60%)	C57BL/6J mice	↑BMAT ↔or ↓ Bone mass(O, μCT)	(174) (175) (161)
Physical exercise (voluntary exercise wheel in NCD and HFD mice)	C57BL/6 mice	↓BMAT volume in NCD and HDF-fed mice. ↑Bone mass (O, μCT)	(176) (63)
Caloric restriction (CR)	C57BL/6J mice (CR: 30% of NCD) New Zealand White rabbits [CR: Moderate (30%) or extensive (50–70%)]	↑ BMA volume (H, MR) ↑BMAT volume (O, μCT, H)	(35) (177)
Acute fasting (48 h)	Sprague-Dawley rats	BMAd size: ↓proximal tibia ↔tail vertebrae (O, μCT)	(178)
Cold exposure (4°C)	C57BL/6J C3H/He	↓rBMAT ↔ cBMAT (O, μCT)	(12)
CLA +FO supplementation	C57BL/6 mice	↓BMAT (H)	(179)
Dietary methionine restriction	C57BL/6J mice	↑BMAT (O, μCT)	(180)

*↓, Decrease; ↑, Increase; ↔, No change; BW, body weight; CLA, conjugated linoleic acid; FO, fish oil; HFD, high fat diet; O, osmium tetroxide staining; BMAT, bone marrow adipose tissue; NCD, normal chow diet; rBMAT, regulated BMAT; cBMAT, constitutive BMAT; BMAd, bone marrow adipocyte*.

**Table 9:** *In vivo* modulation of bone marrow adipose tissue by hormonal and pharmacological treatments in animal models.

***In vivo* intervention**	**Animal model (route of administration)**	**Outcomes (assays)**	**References**
Leptin	*ob/ob* mice (s.c. osmotic pumps)	↓ BMAT volume, ↑ bone formation(H-t)	(181)
	Type 1 Diabetic mice (s.c. osmotic pumps)	↓ Adipocyte number, ↔ bone mass loss(H, μCT)	(182)
	C57BL/6J mice (s.c.)	↓ BMAT formation induced by CR, ↔ BMD(O, μCT, H)	(183)
	Sprague-Dawley rats (VMH injection)	↓ number of BMAT adipocytes(H-t)	(184)
	*ob/ob* mice (rAAV-Lep, i.c.v.)	↓ BMAT, ↑ bone formation(H-f)	(185)
Orchidectomy	C57BL/6J mice	↑ BMAT(H-f)	(152)
PPARγ Agonists *Rosiglitazone* *Ovariectomy (OVX)* *OVX + Rosiglitazone*	Wistar rats (gavage)	↔ Ad.A./M.A., ↑ Ad.A./M.A., ↑ Ad.A./M.A.(H-t)	(186)
Troglitazone	ApoE^−/−^ mice (mixed with diet)	↑ BMAT (Ad.A./M.A.)(H-t)	(187)
Rosiglitazone	C57BL/6J mice C57BL/6J mice + Exercise C57BL/6J mice C3H/HeJ mice DBA/2J mice A/J mice Diabetic yellow agouti Avy/a Ocn-Wnt10b mice (mixed with diet)	*C57BL/6J:* ↑ BMAT (adipocyte number) (H-t) *Exercise:* ↓ BMAT-induced by rosiglitazone (O, μCT) *C57BL/6J:* ↑↑ BMAT (H-f) *C3H/HeJ:* ↑ BMAT (H-f) *DBA/2J:* ↔ BMAT (H-f) *A/J:* ↔ BMAT (H-f)↑ BAT/WAT gene expression in marrow of C57BL/6 mice, not increase BAT genes in diabetic mice (H-t, RT-PCR) ↓ BMAT *vs*. WT (O, μCT)	(188) (189) (190) (7) (191)
PPARγ Antagonists *Bisphenol A Diglycidil Ether, BADGE (partial antagonist properties)*	BALB/c (streptozotocin-induced diabetes) male C57BL/6J mice C57BL/6J mice + lethal irradiation C57BL/6J mice + cytarabine C57BL/6J mice + high fat diet (35%) (i.p)	↓ BMAT, ↔ BMD ↓ BMAT, ↑ BMD ↓ BMAT, ↑ hematopoietic recovery ↔ BMAT, rescue BMD (H-t, μCT, IF, RT-PCR)	(192) (37) (193) (194) (195)
GW9662 (pure antagonist)	C57BL/6 into C.B10-immune BM aplasia (i.p.)	↓ BMAT, ↑ hematopoietic recovery (IF, RT-PCR)	(196)
B-3 Adrenergic agonists *Isoproterenol or CL316,243*	Sprague-Dawley rats (i.p.) C3H/HeJ mice (i.p.)	BMAT from distal tibia and tail vertebrae resists β-adrenergic-induced lipolysis Moderate lipid droplet remodeling of BMAT adipocytes (proximal tibia); (O, μCT, IHC)	(178)
Dexamethasone	C57BL/6J mice (i.p.)	↑ BMAT, ↓BMD(H-f, μCT)	(197)
Lethal irradiation + BM Transplantation	FVB C57BL/6J mice	↑ BMAT ↔ BMAT, ↑ BMD in “fatless” FVB.A-ZIP/F (H-f, μCT) ↑ M.Ad./M.A. ↑ Ad. number and size (MQ-t, f,st,sp & MRI-f)	(193) (61)
	Beagle dogs	↑ BMAT (H-t, hu, r)	(198)
Cytarabine (ARAC) Ablative chemotherapy	C57BL/6J mice	↑ BMAT; (H-t)	(194)

*↓, Decrease; ↑, Increase; ↔, No change; BAT, brown adipose tissue; BMAT, bone marrow adipose tissue; BMD, bone mineral density; f, femur; H, histomorphometry; hu, humerus; IF, immunofluorescence; IHC, immunohistochemistry; i.p., intraperitoneal; O, Osmium tetroxide staining; r, radius; st, sternum; sp, spine; t, tibia; WAT, white adipose tissue; rAAV-Lep, recombinant adeno-associated virus (rAAV)-Leptin; BADGE, Bisphenol A Diglycidyl Ether; BM, bone marrow; CR, calorie restriction; Ad.A., adipocyte area; M.A., marrow area; PPARγ, μCT, micro-computed tomography; RTPCR, real-time quantitative polymerase chain reaction; OVX, ovariectomy; VMH, Ventromedial Hypothalamus; WT, wild-type; s.c., subcutaneous; VMH, ventromedial hypothalamus; i.c.v., intracerebroventricular*.

## *In vivo* BMAT Modulation

### *In Vivo* Lineage Tracing

It is now well-accepted that BMAds differentiate from a small number of radioresistant mesenchymal progenitor cells that reside in the bone marrow. The ability to identify these early progenitor cells, more mature precursor cells, mature marrow adipocytes, and other mesenchymal lineage derived cells (e.g., osteoblasts), has been accomplished by the advent of modern lineage tracing using relatively specific Cre-drivers and fluorescent reporters (5, 84, 137, 199–202). This approach has the added benefit of being able to compare marrow adipocytes to white, brown, and beige adipocytes, and adipocytes in different anatomical locations *in vivo*.

**Figure 5:**
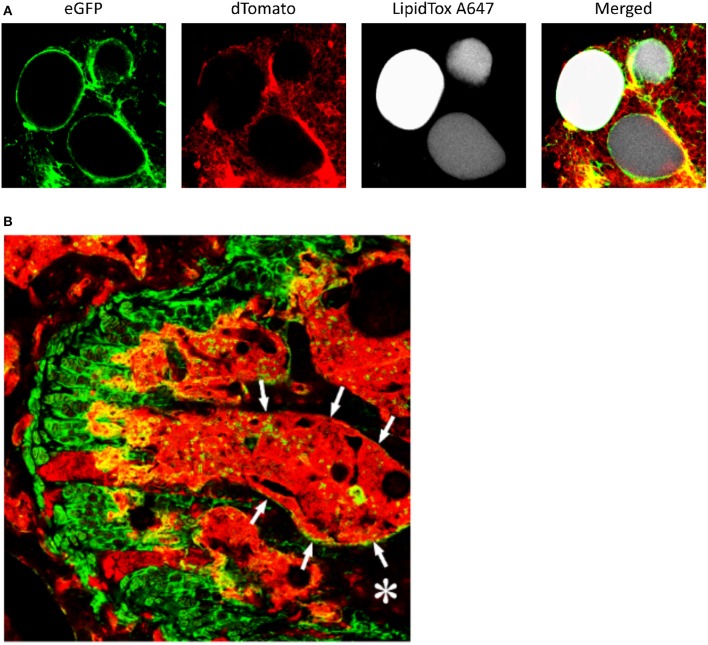
Lineage tracing of bone marrow adipocytes and bone marrow stromal cells. **(A)** Adiponectin-cre:mT/mG mice received a single dose of x-irradiation (1,000 rads) to induce bone marrow adipogenesis. Following irradiation, the mice were reconstituted by an intravenous. injection of 106 syngeneic bone marrow cells to prevent radiation induced bone marrow damage. Bone marrow was collected from the femur as an intact plug, stained with LipidTox (fluorescent lipophilic dye), and marrow adipocytes visualized by confocal microscopy. Greater than 95% of the cells were eGFP^+^ indicating they were traced by expression of Adiponectin. **(B)** The femur from *Twist-2-cre:mT/mG* mice was isolated, the femoral head removed and the bone fixed in 4% paraformaldehyde overnight. The bones were then immersed in 30% sucrose for 3–4 days, then placed in optimal cutting temperature compound, and frozen. Five to 10 μm thick-sections were imaged by confocal microscopy. Columns of growth plate cartilage cells were eGFP^+^. In the bone marrow (outlined by arrows and appearing red), a small number of eGFP^+^ cells can be seen. In addition, approximately 50% of osteocytes (*bone, appearing black) were also eGFP^+^. Cells that were eGFP^+^ were traced by the expression of *Twist-2*.

Today's lineage tracing consistently depends on the Cre/Lox system (203). In the standard Cre/Lox system, Cre recombinase is expressed under the control of a tissue-specific promoter to permanently activate a reporter gene that functions to mark the original Cre-expressing cell population and all daughter cells that develop. Therefore, it is paramount that one has a detailed understanding of the Cre driver's spatiotemporal expression. Lack of this understanding can result in false interpretations of the origin of the cells. As an example, *PdgfR*α*-cre* traces all the adipocytes in white adipose tissue, but within the bone marrow, it traces about 50% of the adipocytes (202). By contrast, *Prx-1-cre* traces all marrow adipocytes (202). It also traces posterior subcutaneous white adipose tissue, including beige adipocyte precursors, as the mesenchymal origin of this depot gets recombined during limb development. In contrast*, Prx1* does not trace the majority of brown adipocytes or any visceral adipocytes (204). Thus, it is useful because of its relative specificity, especially compared to *adiponectin-cre*, which traces all adipocytes including marrow adipocytes (Figure 5A), and a range of BMSC precursors (6). Another advantage to this system are inducible Cres. As an example, *Prx1-ER-cre*, where Cre expression is activated in Prx1 positive cells by tamoxifen injection. These types of constructs allow for the timed induction of the reporter. A caveat to be considered when using tamoxifen and ER-inducible cre-drivers, however, is that the dose of tamoxifen required to efficiently activate Cre expression can be 100–1,000 times greater than required to activate estrogen receptors. A single dose of tamoxifen is reported to have irreversible effects on the uterus. Furthermore, tamoxifen crosses the blood brain barrier to regulate energy metabolism, is a potent immune modulator in mice, and can induce bone marrow failure (205–207). Investigators who choose to use tamoxifen in spite of its potent estrogen receptor-mediated actions, should be advised to include a no treatment control as well as a tamoxifen-treated/no Cre control. Equally important is the nature of the reporter gene used. For example, adipocytes possess little cytoplasm relative to other cell types, therefore cytoplasmic reporters such as LacZ are not optimal for tracing adipocytes. Instead, membrane-targeted reporters such as mT/mG (membrane Tomato/membrane GFP) provide superior results (208, 209). For extensive discussions on this topic see Jeffery et al. (210) and Sanchez-Gurmaches et al. (211).

Once the Cre-reporter has been selected, which will be dictated by the demands of the experiment, either an *ex-vivo* or *in situ* approach can be taken.

The *ex-vivo* approach involves dissecting out the femur, cutting off the femoral head and removing the distal epiphysis above the growth plate. A 20-gauge needle can then be inserted down the medullary canal (from proximal to distal), and punching out the needle through the distal growth plate. Because the distal growth plate is intact this causes the bone marrow to fill the needle. The needle is then attached to a syringe and the marrow plug can then be deposited on a microscope slide by depressing the barrel of the syringe. The adipocytes in the marrow plug can then be prepared for imaging by confocal microscopy. Femora are preferred for this technique because they are fairly uniformly cylindrical along the length of the bone making them amenable to boring.

Advantages: (1) This is a straight forward simple method that requires no specialized equipment, with the exception of the confocal microscope; (2) The method is rapid. It avoids the requirement of decalcification or sectioning; (3) Using *mT/mG* reporter mice, in addition to tracing mature marrow adipocytes, will show whether marrow adipocyte precursors (GFP^+^) expressed the gene of interest; (4) The marrow adipocytes, in the marrow plug, can be stained with a fluorescent lipophilic dye (i.e., LipidTOX) allowing for easy identification of the mature adipocytes. This also allows for better determination of cell counts and size. This approach can be combined with immunofluorescence to co-stain for other cell markers if desired.

Challenges: (1) Because adult mice (C57BL/6 background) have few marrow adipocytes in the femur, induction of marrow adipogenesis is recommended. However, the choice of which induction protocol to be used (x-irradiation, high fat diet feeding, feeding with a methionine restricted diet or a rosiglitazone containing diet, see Tables 8, 9) will depend on the experimental design; (2) The femoral medullary canal in adult mice is the only site sufficiently large to collect a workable bone marrow plug; (3) Because the cells are removed from the marrow their anatomical location, especially as it relates to trabecular bone and the endosteum is lost.

The second, *in situ*, approach maintains anatomical location with respect to the growth plate and endosteum, but by maintaining the calcified bone matrix, introduces its own complications. Although the *in situ* approach involves collecting fresh femurs, from that point, the method varies significantly from investigator to investigator. The bones can be fixed in paraformaldehyde overnight and then given a partial decalcification in EDTA, sucrose incubation follows, and then embedding in either a cryomedia or carboxymethyl cellulose, followed by frozen sectioning (Figure 5B). Some of the best images have been acquired using a tape-transfer system and cutting 10–30 μm sections (137, 199–201). Other investigators have even used paraffin embedding instead of frozen sections, although this necessitates the use of antibodies, even in fluorescence reporter mice (5). After sectioning, the tissue can then be stained with the desired antibody-conjugate (direct i.e., GFP, or indirect using a secondary fluorescent antibody or biotin-avidin conjugate), and imaged by fluorescence or confocal microscopy. However, the fixation and decalcification can vary greatly form investigator to investigator, including some who use no fixation and rapid freezing (200). In addition, to immunofluorescent staining, transient fluorescent reporter mice (e.g., *Zfp423-EGFP*) or Cre/Lox lineage tracing fluorescent reporter mice can be used.

Advantages: (1) The major advantage to this method is that it allows for direct visualization of the cells within intact bone. Thus, the spatial relationship between marrow adipocytes, other cells, and bone is maintained; (2) Using *mT/mG* reporter mice can be a significant advantage; (3) Mature marrow adipocytes can be imaged.

Challenges: (1) This method requires expertise and experience in bone histology and specialized histologic equipment (e.g., tungsten-carbide knifes to section bone); (2) Sectioning small bones (e.g., distal tibia and caudal vertebrae) can be difficult; (3) Difficulties using the tape-transfer systems have been reported; (4) The embedding techniques and section preparation often exclude the combined use of lipid-tracing dyes.

It is clear that great progress in lineage tracing of marrow adipocytes has been made during the last few years, due to advances such as the Cre/Lox system. Our ability to delineate cells in the bone marrow adipocyte lineage will only get better with the advent of more specific Cre-drivers and more robust reporters. Refinements in our ability to process bone to make it more accessible to these methods will result in an even better understanding of the lineage, how it relates to other mesenchymal lineage cells, and the myriad of other cells in bone marrow.

### *In vivo* Modulation of BMA

BMAT is a complex and dynamic depot that is highly regulated and can affect the function of other tissues/organs. Whether presence of BMAT is necessary for normal physiological responses is still controversial. While some studies have shown that BMAT negatively influences bone mass, a study in BMAT-deficient Kit^W/W−v^ (BMAT^−^) mice suggested that the absence of BMAT did not have any relevant effect on ovariectomy-induced bone loss (22). However, a recent study in BMAT^−^ male mice has shown that absence of BMAT exacerbated bone loss during hindlimb unloading (212).

The expandability of BMAT is regulated by nutritional and environmental factors, aging, endocrine signals, and pharmacological agents. Here, we critically summarize experimental models used to study *in vivo* regulation of BMAT development and function.

#### Nutritional and Environmental Interventions

In C57BL/6J mice, a strain susceptible to obesity and diabetes, HFD feeding induces also BMAT expansion (174, 175). When diet-induced obesity (DIO) is reversed by switching to normal chow diet (NCD) to mimic weight loss, the HFD-induced BMAT recedes (175). Some of these alterations are microbiota-dependent (195). The alterations induced by the HFD on BMAT gene expression differ from that observed in peripheral adipose tissues. In contrast to visceral WAT, pro-inflammatory gene expression was decreased while the expression of genes of the insulin signaling pathway increased in BMAT of HFD-fed mice, suggesting a differential metabolic regulation of BMAT adipocytes (161). Walji et al. (213) used microfibril-associated glycoprotein-1 (MAGP1) deficient (*Mfap2*^−/−^) mice that develop adult-onset obesity that precedes insulin resistance. In these mice, BMAT increased relative to WT mice coincident with the development of insulin resistance, and not with excess peripheral adiposity, hyperglycemia, change in trabecular bone volume or hematopoiesis.

Exercise is a life-style intervention proposed to prevent/counteract obesity-associated BMAT expansion (Table 8). Voluntary wheel running in C57BL/6 fed NCD or HFD demonstrated that exercise prevented the increase in BMAT acquisition (176). Styner et al. (189) found that exercise (alone or in combination with rosiglitazone) reduced BMAT volume and upregulated UCP1 expression in whole tibia (Table 9). Exercise can also reverse the increase of BMAT observed in previously obese animals (HFD-fed for 3 months) by decreasing both adipocyte number and size (63). Exercise was associated with higher trabecular and cortical bone quantity in lean and obese mice, but HFD itself did not influence bone quantity. Importantly, a recent study, also provided evidence that physical exercise modulates vertebral BMAT in humans (214).

The differential *in vivo* regulation of BMAT by nutritional status also occurs in animal models of caloric restriction (CR). In contrast to what is observed in visceral or subcutaneous WAT, BMAT is preserved or even increased in states of CR [(35, 177, 178, 183); Tables 8, 9]. Indeed, CR (30%) in young growing mice alters bone formation, but despite having a lower body weight and body fat percentage, they exhibit a dramatic increase in BMAT (35). In patients with anorexia nervosa, CR is also associated with increased BMAT (215, 216). However, in New Zealand rabbits moderate or extensive CR did not cause BMAT expansion (177). It has been suggested that the increase in BMAT is especially prominent when nutrient deprivation occurs during periods of skeletal growth, such as childhood or adolescence (178). This period of rapid skeletal growth may already be poised for BMAT development as this is also a time of rapid baseline BMAT accumulation (217).

The expansion of BMAT during CR has also been associated with changes in several neuroendocrine factors that are modulated in response to energy deprivation. The decrease in leptin that occurs during CR-induced weight loss may account for the increased BMAT. Indeed, BMAT is increased in leptin-deficient *ob/ob* mice (218), and subcutaneous leptin treatment induces loss of BMAT adipocytes and increases bone formation in these mice (181). Moreover, peripheral leptin therapy is effective in reversing the increased BMAT observed in type 1 diabetic mice and CR models, but does not stop the bone loss that occurs concomitantly [(182, 183); Table 9]. Furthermore, central injections of leptin into the ventromedial hypothalamus (VMH) of Sprague-Dawley rats, as well as leptin gene therapy (intraventricular administration of recombinant adeno-associated virus (rAAV)-leptin gene) to *ob/ob* mice also reduced BMAT (184, 185). Interestingly, mice with selective deletion of the leptin receptor (Lepr) in limb bone marrow stromal cells (Prx1-Cre;Lepr^fl/fl^ mice) exhibited normal body mass and hematopoiesis, but have decreased BMAT, and increased osteogenesis (219). Moreover, Prx1-Cre;Lepr^fl/fl^ mice were protected from the HFD-increases in BMAT and reductions in osteogenesis. It therefore appears that hypothalamic and peripheral leptin signaling may have different or multiple effects on adipogenesis within bone marrow.

#### Aging

Increased BMAT is also observed during aging, and has been negatively correlated with bone health, and sometimes precipitates impaired hematopoiesis in animals (5, 220) and humans (221–223). Dietary strategies have also been proposed to counteract the increased BMAT associated with aging, and combination of conjugated linoleic acid with fish oil can decrease age-associated BMAT in C57BL/6J mice (179). Dietary methionine restriction (MR) increases longevity in rodent models, however MR promotes BMAT accumulation in contrast to WAT reduction (180).

#### Endocrine Regulation

From an endocrine perspective, bone and BMAT metabolism are tightly linked and therefore BMAT is under extensive hormonal regulation. First of all, already a long time ago it has been observed that ovariectomy increases BMAT in animals (224) and ovariectomy is now commonly used to induce BMAT in animal models. These observations have been extended to humans, as BMAT increases during aging and this increase is accelerated in women around the time of menopause (225). Post-menopausal hormonal replacement therapy with estradiol, both long term (1 year) and short term (2 weeks) decreases BMAT in women (33, 226), showing that indeed estradiol is an important regulator of BMAT. At the same time that estradiol secretion by the ovaries ceases, compensatory follicle stimulating hormone (FSH) secretion by the pituitary gland increases. In addition to the effect of hormonal replacement therapy, also FSH blocking therapy has been shown to decrease BMA in mice (83). In addition to gonadal hormones, glucocorticoids have a profound effect on adipose metabolism and this also holds true for bone marrow adiposity. Cushing's disease, defined by increased adrenocorticotropic hormone (ACTH) production by a pituitary adenoma and therefore hypercortisolemia, increases BMAT and this reverses again following surgical cure by removal of the pituitary adenoma (227). Also, long-term glucocorticoid treatment leads to increased BMAT (228) and can be used to induce BMAT in animal models. Finally, parathyroid hormone, an important regulator of bone metabolism and potent osteoanabolic drug, also has an effect on BMA. Teriparatide treatment in osteopenic women reduces BMAT (229) and animal studies showed that this effect can be recapitulated by genetic deletion of the parathyroid hormone receptor in skeletal stromal cells (56). Interestingly, additional studies from the Rosen lab showed that the effect of PTH is not only on the differentiation of the SSC into the adipocytic lineage, but that Parathyroid Hormone (PTH) can also induce lipolysis in BM adipocytes (230). In addition, growth hormone (GH) is an important regulator of skeletal growth and growth hormone deficiency or resistance has been associated with changes in BMAT. In growing rats, hypophysectomy dramatically increases BMAT and this could not be reversed by treatment with either estradiol, thyroid hormone, cortisol or Insulin Growth Factor-1 (IGF-1), but was completely reversed by treatment with GH (231). In healthy, premenopausal women, vertebral BMAT measured with 1H-MRS was inversely associated with IGF-1 concentrations, but not stimulated GH concentrations (232). However, treatment with recombinant GH for 6 months in premenopausal obese women, did not change BMAT, although there was a significant difference between the GH treated and placebo treated groups due to the decrease in BMAT in the placebo group (233). Therefore, the role of GH in the regulation of BMAT in adult humans remains uncertain and studies in children during growth have not been performed.

BMAT is not only regulated by hormones, but also acts as an important endocrine organ itself. Cawthorn et al. (234) found that increased BMAT significantly contributes to the higher circulating adiponectin levels during CR. Moreover, studies in Ocn-Wnt10b mice, which resist BMAT expansion during CR, demonstrated that increased BMAT is required for the elevated circulating adiponectin in this condition. Furthermore, BMAT and adiponectin levels increase in patients undergoing therapy for ovarian or endometrial cancer, despite no change in total fat mass (234). Increased adiponectin levels and BMAT volume were also observed in DIO WT (C57BL/6J) mice treated with Rosiglitazone. However, female Ocn-Wnt10b mice treated with Rosiglitazone had mildly blunted hyperadiponectinemia (191) while males did not, suggesting a sex-specific response.

#### Pharmacological Modulation

Several drugs also regulate BMAT. PPARγ is a master transcription factor for adipocyte differentiation, and treatment with the insulin-sensitizing drugs thiazolidinediones (TZDs), which are PPARγ agonists, affects marrow adiposity. As shown in Table 9, treatment with several PPARγ agonists such as Rosiglitazone and Troglitazone enhanced BMAT in different animal models (187, 188). However, the effects of TZDs on BMAT seem to be strain-specific (190) and age-dependent, favoring BMAT accumulation in older mice rather than in young-growing animals (188). In ovariectomized (OVX) rats, treatment with rosiglitazone (BRL49653) exacerbated bone loss and increased BMAT (186). On the other hand, several studies have shown that treatment with PPARγ agonists increased BMAT without affecting trabecular bone volume, suggesting that adipogenesis and osteogenesis can be regulated independently *in vivo* (187). Similarly, netoglitazone administered to 6-month-old C57BL/6 mice had a strong adipogenic induction with no change in the trabecular architecture and modest decreases in cortical bone mineralization (235). In contrast, a reduction in BMAT has been observed in all studies administering PPARγ antagonists after chemo/radiotherapy, which are potent inductors of BMAT [(37, 192–194, 196, 236); Table 8]. Moreover, genetic models of PPARγ loss show a pronounced increase in bone mass with extramedullary hematopoiesis (237, 238). The effects of BMAT in the recovering of hematopoietic compartment seem apparently contradictory, possibly due to the differential effects of distinct BMAd subtypes and differentiation stages in hematopoietic progenitor support [reviewed in (239, 240)]. Methodologically, it is to be noted that although effective in reducing BMAT, the most commonly used PPARγ “antagonist” for *in vivo* experimentation, Bisphenol A Diglycidyl Ether (BADGE), has partial PPARγ agonist effects and is a potential endocrine disruptor receptor anti-androgenic and pro-estrogenic properties (241–243). It is therefore recommended that future *in vivo* studies use a more specific PPARγ antagonist such as GW9662 (196).

BMAT thus accumulates following hematopoietic marrow ablation. A wave of BMAT precedes hematopoietic repopulation and peaks from 2 to 3 weeks after whole-body radiation depending on dose and recipient characteristics (700–1,000 Gy) (193, 202). BMAT is then lost and the timing of recovery depends on the radiation dose and on the number of hematopoietic cells used for the rescue. Sublethal models which do not require hematopoietic rescue have also been developed with 5-fluorouracil or cytarabine treatments (194, 236). Both radiation and chemotherapeutic treatments induce dramatic increases in BMAT also in patients (234, 244), whereas certain disorders of inefficient hematopoiesis (e.g., W/Wv mice) are associated with greatly reduced BMAT and a modified lipid composition of the stroma (245, 246). The biological implications of BMAT in neoplastic progression within the BM microenvironment are only beginning to unravel (247–251).

#### Sympathetic Regulation

A very important issue when studying the *in vivo* modulation of BMAT is to consider the region-specific variation in the properties of the skeletal adipocytes, as already discussed in the BMAT isolation section. The studies of Scheller et al. (12) in mice strongly support the existence of a constitutive (cBMAT) and a regulated (rBMAT) depot. cBMAT is in the distal long bones fills the medullary canal from the tibia-fibular junction into the malleolus and caudal vertebrae, histologically resembles WAT, appears rapidly in the early postnatal period, does not usually respond to stimuli or pathophysiological changes (202), though it can reduced over several months with thermoneutrality (252). In contrast, rBMAT is situated in the proximal regions of long bones and in spinal vertebrae, develops after cBMAT, and is interspersed with hematopoietic cells. rBMAT increases or decreases in various conditions (DIO, aging, CR, etc.) (253).

The study of Scheller et al. (12) also revealed that knockout of PTRF (Ptrf^−/−^ mice, a model of congenital generalized lipodystrophy 4) selectively inhibits formation of rBMAT adipocytes without affecting cBMAT, which could be one step toward generation of a genetic model of rBMAT ablation (12).

The lack of response of BMAT adipocytes to lipolysis during energy deprivation has been attributed to resistance to β-adrenergic stimulation, but this effect also shows region-specific differences. Acute fasting (48 h) decreases cell size of BMAT adipocytes within the proximal tibia but not within the tail vertebrae in Sprague-Dawley rats (178). Moreover, BMAT from distal tibia and tail vertebrae of these rats resists β-adrenergic-induced phosphorylation of Hormone-Sensitive Lipase (HSL) and/or perilipin, which are required for stimulation of lipolysis. Furthermore, treatment of C3H/HeJ mice with CL316,243, a β3-AR agonist, caused remodeling/beigeing of WAT, but only moderate remodeling of lipid droplets in BMAT of proximal tibia without affecting mid or distal tibia (Table 9). Furthermore, β-adrenergic stimulation through cold exposure shows lipolytic response by rBMAT (decreased rBMAT in the tibial epiphysis and in the proximal tibia) while cBMAT remained unchanged (12). Therefore, these data suggest that the lipolytic response to β-adrenergic stimulation is more pronounced in rBMAT than in cBMAT (178).

Another important factor to take into account when designing and interpreting studies about *in vivo* BMAT regulation is the effect of housing temperature. Most of the studies in mouse models are performed at room temperature (RT, around 22°C), which is below the thermoneutral temperature for mice (around 32°C). Therefore, RT housing can increase non-shivering thermogenesis by the sympathetic outflow and the activation of UCP1 in BAT (254, 255) showed that thermoneutral housing not only reduces UCP1 expression in BAT, but also increase BMAT and the percentage of body fat as compared with RT-housed mice. Therefore, the mild cold stress induced by RT-housing could be a non-considered confounding factor in mice studies.

#### *In vivo* Modulation Challenges

All these studies have demonstrated that BMAT expansion accompanies metabolic dysfunction. However, many physiopathological changes take place in these processes, and dissecting the role of BMAT expansion from the role of peripheral adipocytes and other metabolic perturbations make mechanistic studies a challenge. Furthermore, the divergent BMAT responses to different strains/species suggest the existence of a strong genetic background effect, which should be considered when designing studies, highlighting once more the importance of adhering to the BMAS minimal reporting guidelines when communicating results (see Table 1). Possibly, standard *in vivo* experimental conditions need to be defined for inter-laboratory comparisons. As we continue to identify the physiological processes that underlie the formation of BMAT and the environmental and genetic cues that control its accumulation, it is becoming increasingly evident that BMAT may be heterogenous.

Finally, in spite of their limitations, wider use of available genetic models of non-selective BMAT depletion (e.g., Ptrf^−^/^−^, W/Wv), which have lesser metabolic phenotypes than severely lipodystrophic mice (e.g., A-ZIP/F), should advance the field until models of highly-specific BMAT depletion can be developed. Furthermore, it is paramount to consider that the BMA, bone, vascular and hematopoietic compartments are tightly interlinked within the BM, such that *in vivo* analysis requires functional measurements of all four compartments to reach mechanistic conclusions.

## Biobanking

The BMAS Working Group on Biobanking has the ambition to generate standardized approaches toward isolation, characterization and long-term storage of tissues/cells related to BMA and their associated data and annotations. Although difficult to achieve due to several challenges (see below), creating minimal standards to isolate and characterize BMAds as well as freezing protocols for long-term storage should significantly reduce variability in outcomes between studies and laboratories. This is especially important to ensure viability and conservation of heterogeneity in cell-based assays, and to ensure sample stability for chemical analysis including mass spectrometry. Most importantly, unified biobanking standards along with the protection of associated data will enable responsible use and exchange of samples for comparative and larger-scale studies. Ultimately, the field will benefit from improved applicability of animal and human BMA-related samples, which may better facilitate the discovery of novel therapeutics to target BMA.

In a complementary fashion to the BMAS Working Group in Methodologies, one of the foci of the BMAS Biobanking Working Group will be to congregate methodologies related to the collection, freezing/thawing and long-term storage of BMAds. Additional aspects of biobanking that the working group will scrutinize are privacy regulations regarding participants/patients, data protection and ethical guidelines to facilitate collaborative efforts. Main challenges toward this objective are briefly introduced below.

### Isolation of BMAds

Different types of materials have been and will be employed to study BMA, including BM aspirates, biopsies, specific cell types, BM plasma fraction, etc. After having defined these, recommended standard procedures are required regarding BMA isolation, processing and characterization. For example, isolation protocols vary substantially between laboratories (digestion with collagenase or other enzymes, incubation times, etc.). In addition, it is important to distinguish protocols for animal studies vs. human materials as BMA is different in composition, location, metabolism and regulation. For human bone marrow–related samples, recommended patient screening should be additionally established (HIV, Hepatitis B and C virus). Finally, minimal standards should also be established for sample annotation, which should include description of the site of collection, method of collection and isolation, including time and type of digestion, time from collection to freezing, etc.

### Characterization of BMAds

One of the biggest challenges is to define a healthy control set, especially for human samples (see Table 1). What is regarded as normal population and a standard site of collection and how do we define this? What is the minimal set of parameters required to define such a set? One solution is to propose a minimal set of specific surface molecules, gene expression markers and/or other biomarkers (e.g., lipid profiles) to facilitate comparisons and thus also multicenter studies. Some specific markers have been proposed (adipokine markers, absence of hematopoietic, endothelial, and hematopoietic markers) but additional species-specific markers are needed to be able to characterize BMAds in a uniform and reliable fashion.

### Long-Term Storage of BMA-Related Samples

To date it has proven impossible to freeze BMAds, and the only access point to retrospective samples relies on the identification of adipocyte ghosts in paraffin blocks. Tissue samples containing adipocytes are being collected but these require purification and/or digestion steps before freezing. On the other hand, storage of precursor cells (SSCs) may poorly reflect the BMA situation at the time of isolation, most often due to cellular expansion (and deviation) *in vitro* before or after freezing. In order to create as much homogeneity as possibly, it is vital to define freezing and thawing procedures employed with a minimum of interfering steps as well as viability and cell growth/differentiation characteristics of previously stored BMA samples. Non-frozen samples, including paraffin blocks, may pose less issues, but still homogeneity in tissue processing and database management are required to optimize storage and exchange of samples.

### Ethical Issues and Data Protection

With the installment of the General Data Protection Regulation (GDPR) in 2018 (EU GDPR Portal (website), accessed August 27, 2019, http://eugdpr.org) the protection of people and data has become more stringent. Trying to come up with a standardized procedure toward biobanking will face challenges, including different national regulations, institutionalized rules, etc. Ethical guidelines varying between countries should be dealt with to assess the possibility to generalize ethical topics into one document for samples to be collected in the future. Related to this, a general template for informed consent and awareness of mutual use of obtained samples by all involved may improve standardization and the possibility to share samples, which is especially relevant in the context of rare diseases. A BMAS consortium-wide material transfer agreement may be instrumental for this.

Issues related to data protection include the assurance that participant/patient data remains anonymous at all costs. Although the consciousness around this subject is increasing, the working group will assess these issues in detail and will aim to propose a comprehensive recommended protocol to safeguard anonymity and data protection that originates from any of the laboratories. Data obtained in the European union (EU) often cannot be stored on servers outside the EU and similar regulations may apply to different continents as well. Therefore, a robust data management plan needs to be installed that can explore and potentially overcome challenges such as decentralized storage of samples and associated files.

In conclusion, biobanking, and methodological challenges are tightly linked. Minimal standards and international overarching ethical guidelines for BMA sample collection and data protection will be critical to increase the quality of fundamental and multicenter clinical studies, and interpret with greater confidence the outcome and impact of BMA research within the next few years.

## Concluding Remarks

Specific methodologies for the study of BMA have been developed in the last decade, paralleling the increasing interest in the field. Gold standard methodologies currently exist for the assessment of BMA *ex vivo, in vivo* and *in vitro* (e.g., histomorphometry and OsO_4−_ 3D contrast-enhanced μCT for *ex vivo*, WFI and 1H-MRS MRI sequences for *in vivo* studies, lipid-dye-based and RT-qPCR-based assessment for assessment of *in vitro* BM adipogenesis) and emerging techniques may soon come to complement or substitute these gold standards (i.e., digital pathology algorithms for histomorphometry, POM-based contrast-enhanced CT for *ex vivo* imaging, dual energy CT for *in vivo* imaging as well as more reproducible parameters for *in vivo* MRI spectroscopy, label-free or 3D microscopy and microspectroscopy for *in vitro* imaging, or 3D adipose organoids for *in vitro* cultures).

However, great challenges still remain. First, given the inherent fragility of BMAds and their difficult access within the bone, protocols for extraction, *ex vivo* handling, and *in vitro* culture/differentiation of BMAds or BMSC progenitors vary greatly. Thus, recommended standardized protocols for *in vivo* modulation and extraction, minimal standards for BMAd purity assessment, and standardization of method-specific thresholds for BMA detection would greatly increase inter-study comparison and multi-site collaborations. Second, given the number of factors that affect BMA mass, and possibly type (e.g., skeletal location, gender, age, strain, nutritional status, metabolic state, exercise, ambient temperature, isolation technique), great attention needs to be paid in careful annotation and reporting of these confounding factors for all BMA scientific output. Third, in order to move forward the functional understanding of BMA, tools for the specific ablation of BMAds are urgently needed to uncouple the local BMA-effects from the metabolic effects of systemic lipodystrophy.

The BMAS Working Group in Methodologies and the collaborative BMAS community at large present the opportunity to reach methodological consensus guidelines and propose minimal standards that would strengthen the quality of our scientific output, increase comparability and prepare the field for multi-site preclinical and clinical studies which can pave the way to sound clinical translation. As a first step, incorporation of the BMAS nomenclature guidelines presented in the accompanying piece of this issue (31) and adherence to the methodology reporting guidelines presented here (Table 1) will ensure a common language for our community. In addition, we would like to invite readers to contribute by commenting on this review in the comments section of the “Frontiers in Endocrinology” website.

## Author Contributions

JT, AV-V, and ON conceptualized the manuscript, coordinated the writing, and edited the sections. JT, BP, RL, and PB mounted the manuscript and tables. RL, NB, JT, and AV-V wrote the histomorphometry section. GK, ED, and ES wrote the *ex vivo* imaging section. SB and DK wrote the *in vivo* imaging section. JT, SB-C, BP, AP, PB, and ON wrote the *in vitro* section. MM-A, JF, AV-V, and ON wrote the *in vivo* modulation section. JF, MR, CR, and MH wrote the *in vivo* reporter section. ON and BE wrote the biobanking section. All authors edited and approved the final version of the manuscript.

### Conflict of Interest

AV-V and ON are co-chairs and JT is coordinator of the BMAS Working Group in methodologies. BP, BE, CR, ED, and ON are members of the BMAS Executive Board. AV-V, DK, ES, GK, and MH are members of the BMAS Scientific Board. The remaining authors declare that the research was conducted in the absence of any commercial or financial relationships that could be construed as a potential conflict of interest.
